# Genome-wide identification and expression analysis of calmodulin and calmodulin-like genes in passion fruit (*Passiflora edulis*) and their involvement in flower and fruit development

**DOI:** 10.1186/s12870-024-05295-y

**Published:** 2024-07-03

**Authors:** Dan Zhang, Lumiao Du, Jinting Lin, Lulu Wang, Ping Zheng, Biao Deng, Wenbin Zhang, Weiqiang Su, Yanhui Liu, Yuming Lu, Yuan Qin, Xiaomei Wang

**Affiliations:** 1 Horticulture Research Institute, Guangxi Academy of Agricultural Sciences, Nanning Investigation Station of South Subtropical Fruit Trees, Ministry of Agriculture, Nanning, 530007 China; 2 College of Life Science, Fujian Provincial Key Laboratory of Haixia Applied Plant Systems Biology, Pingtan Institute of Science and Technology, Fujian Agriculture and Forestry University, Fuzhou, 350002 China; 3 Fine Variety Breeding Farm in Xinluo District, Longyan, 364000 China; 4 College of Life Sciences, Longyan University, Longyan, 364000 China

**Keywords:** Calmodulin, Calmodulin-like, Passion fruit, Flower and fruit development

## Abstract

**Background:**

The calmodulin (CaM) and calmodulin-like (CML) proteins play regulatory roles in plant growth and development, responses to biotic and abiotic stresses, and other biological processes. As a popular fruit and ornamental crop, it is important to explore the regulatory mechanism of flower and fruit development of passion fruit.

**Results:**

In this study, 32 *PeCaM*/*PeCML* genes were identified from passion fruit genome and were divided into 9 groups based on phylogenetic analysis. The structural analysis, including conserved motifs, gene structure and homologous modeling, illustrates that the PeCaM/PeCML in the same subgroup have relative conserved structural features. Collinearity analysis suggested that the expansion of the *CaM*/*CML* gene family likely took place mainly by segmental duplication, and the whole genome replication events were closely related with the rapid expansion of the gene group. *PeCaM*/*PeCMLs* were potentially required for different floral tissues development. Significantly, *PeCML26* had extremely high expression levels during ovule and fruit development compared with other *PeCML* genes, suggesting that *PeCML26* had potential functions involved in the development of passion fruit flowers and fruits. The co-presence of various *cis*-elements associated with growth and development, hormone responsiveness, and stress responsiveness in the promoter regions of these *PeCaM*/*PeCMLs* might contribute to their diverse regulatory roles. Furthermore, *PeCaM*/*PeCMLs* were also induced by various abiotic stresses. This work provides a comprehensive understanding of the *CaM*/*CML* gene family and valuable clues for future studies on the function and evolution of *CaM*/*CML* genes in passion fruit.

**Conclusion:**

A total of 32 *PeCaM*/*PeCML* genes were divided into 9 groups. The *PeCaM*/*PeCML* genes showed differential expression patterns in floral tissues at different development stages. It is worth noting that PeCML26, which is highly homologous to AtCaM2, not only interacts with multiple BBR-BPC TFs, but also has high expression levels during ovule and fruit development, suggesting that *PeCML26* had potential functions involved in the development of passion fruit flowers and fruits. This research lays the foundation for future investigations and validation of the potential function of *PeCaM*/*PeCML* genes in the growth and development of passion fruit.

**Supplementary Information:**

The online version contains supplementary material available at 10.1186/s12870-024-05295-y.

## Background

As the main second messenger in plant cells, calcium plays an important role in regulating plant signal transduction [1]. It is involved in regulating plant stress response, growth and development, programmed cell death and other cell processes [2]. A large number of environmental and developmental signals can cause rapid and transient changes in intracellular free Ca^2+^ concentration [3]. Calcium-binding protein acts as a Ca^2+^ sensor to detect and decode calcium signals, thereby activating downstream reactions [4]. In plants, there are four main types of Ca^2+^ sensors: calmodulins (CaMs) and calmodulin-like proteins (CMLs), calcineurin B-like proteins (CBLs) and calcium-dependent protein kinases (CDPKs) [5–7]. Most Ca^2+^ sensors have been found to have a conserved EF-hand (helix-loop-helix structure), which is a Ca^2+^ binding site [8]. Ca^2+^ binding leads to changes in protein conformation, which in turn interacts with downstream target proteins and affects their activity, forming a complex calcium signal transduction network system, eventually leading to changes in specific cellular physiological processes [9]. Previous studies have shown that CaM is a highly conserved calcium-binding protein that is widely present in all eukaryotes and contains four EF-hand; CML only exists in plants and some protists, containing 1–6 EF-hand [10, 11]. Each EF-hand contains two α-helices connected by a 12-amino acid loop region [11]. CaM and CML have no catalytic activity, but act as sensor relays to regulate downstream targets [11].

As important Ca^2+^ sensors, CaMs and CMLs are widely involved in plant growth and development at various stages [12]. For example, *AtCML23* and *AtCML24* can inhibit the expression of *FLOWERING LOCUS C* (*FLC*) gene, thereby affecting the autonomous regulation pathway of the transition to flowering [13]. Moreover, *AtCML24* and *AtCML25* play a crucial role in pollen germination and pollen tube elongation [14, 15]. *AtCML39* plays a vital role in promoting the transduction of light signals during early seedling establishment in *Arabidopsis*. Besides, overexpression of *TaCML20* enhanced the accumulation and yield of water-soluble carbohydrates in wheat [16]. Cotton *GhCaM7* promotes cotton fiber elongation by regulating the production of reactive oxygen species (ROS) [17]. In addition, CaMs and CMLs also play an important role in plant response to biotic and abiotic stresses [12, 18]. Overexpression of *AtCML43* in *Arabidopsis* enhances sensitivity and can play a role in the plant’s immune response to pathogens [19]. *AtCML20* negatively regulates ABA and drought stress responses in *Arabidopsis* guard cells [20], while *AtCML37* positively regulates ABA accumulation induced by drought stress [21]. Overexpression of *OsMSR2* (rice *CML* gene) can enhance the salinity tolerance, drought tolerance, and ABA sensitivity in *Arabidopsis* [22]. Overexpression of *GmCaM4* in soybean can enhance resistance to plant pathogens and improved tolerance to salt stress [23].

Passion fruit (*Passiflora edulis*) is a woody vine of the Passifloraceae family which is widely distributed in tropical and subtropical regions [24]. It enjoys the reputation of “the king of juice” due to its attractive aroma of juice that has more than ten kinds of fruits, including mango, pomegranate, banana, strawberry, lemon, pineapple, and litchi [25]. Previous studies have shown that passion fruit is rich in essential vitamins, amino acids, flavonoids, alkaloids, and other bioactive components [26]. Due to the good taste and great nutritional benefits, passion fruit is highly appreciated for fresh consumption and industrial purposes [25, 27]. Additionally, many varieties of passion fruit also have important ornamental value with colorful flowers which characterized with large floral tissues and bright coronal filaments. Moreover, passion fruit is susceptible to environmental factors such as temperature, light, and water, which will seriously affect the flowering, yield and fruit quality of passion fruit [28]. Primary and secondary metabolism processes are closely related to the formation of flower appearance, fruit flavor and quality, as well as stress responses [29]. Thus, it is of great significance to explore the regulatory mechanism of growth and development, and stress responses for maintaining the economic benefits of passion fruit.

Systematic identification and investigations of the *CaM*/*CML* genes in various species would further increase our understanding of their evolution and functions, which has been performed in many model plants and crops, such as *Arabidopsis* [30], rice (*Oryza Sativa* L.) [31], grapevine (*Vitis amurensis*) [32], tomato (*Solanum lycopersicum*) [33], apple (*Malus* × *domestica*) [34], Cucumber (*Cucumis sativus* L.) [35]. However, the *CaM*/*CML* gene has not been systematically identified and analyzed in passion fruit, an important tropical fruit crop. Recently, the genome sequence of passion fruit has been published [36, 37], which provides a good opportunity to identify the *CaM*/*CML* gene family members and explore their potential regulatory roles in flower and fruit development and stress responses of passion fruit. Here, a total of 32 *PeCaM*/*PeCML* genes were identified, and a series of systemic analysis were also performed including the phylogenetic relationships, gene structure, conserved motifs, protein-protein interaction network and expression pattern analysis. Interestingly, we found that although most *PeCML* genes were expressed at different developmental stages of flowers and fruits, *PeCML26* had extremely high expression level during ovule and fruit development compared with other *PeCML* genes, indicating that *PeCML26* may be involved in the flower and fruit development of passion fruit. Furthermore, we also analyzed the response of *PeCaM*/*PeCMLs* under different abiotic stresses. Our work could contribute to lay a foundation for further functional investigation on the *CaM*/*CML* gene family in passion fruit.

## Materials and methods

Identification and characterization of the *PeCaM*/*PeCML* genes in passion fruit.

The passion fruit (*Passiflora edulis*) genomic data was retrieved from the National Genomics Data Center (NGDC) (https://ngdc.cncb.ac.cn/gwh/Assembly/17982/show, accession number: GWHAZTM00000000) [38]. The hidden Markov Model profile (HMM) of the EF-hand_7 (PF13499) was obtained from the Pfam server (https://www.ebi.ac.uk/interpro/entry/pfam), which was used as the seed model for the HMMER search of candidate *PeCaM*/*PeCML* genes from the passion fruit genome in TBtools (v1.120) [39], with a cut off E-value of 1 × 10^− 5^. The redundant sequences were removed manually. The Simple Modular Architecture Research Tool (SMART) (http://smart.embl-heidelberg.de/) [40] and the NCBI-Conserved Domains Database (CDD) (https://www.ncbi.nlm.nih.gov/cdd) [41] database were used to cross-check the presence of the EF-hand domains in candidate sequences. Finally, the sequence containing only the EF-hand domain was retained as the potential PeCaM/PeCML member.

The physicochemical feature of the potential PeCaM/PeCML protein sequences, including molecular weight, theoretical isoelectric point (pI), and the number of amino acids was further analyzed using ExPASy (https://www.expasy.org/) [42]. In addition, the subcellular localization of deduced PeCaM/PeCMLs was predicted through the online website Cell-PLoc 2.0 (http://www.csbio.sjtu.edu.cn/bioinf/Cell-PLoc-2/) [43].

Phylogenetic Relationships of CML Proteins.

Then the *Arabidopsis* CaM and CML protein sequences were downloaded from the *Arabidopsis* Information Resource (TAIR) (http://www.Arabidopsis.org/). The CML protein sequences of *Oryza sativa* L., and *Populus trichocarpa* were obtained according to relevant literatures [31, 34, 44]. A neighbor-joining (NJ) phylogenetic tree was constructed by MEGA (version 11.0) [45] based on the alignment of the CaM/CML protein sequences of passion fruit and *Arabidopsis*. The parameters were set as follows: bootstrap analysis with 1,000 replicates, poisson correction, and pairwise deletion. In addition, we also constructed a phylogenetic tree of CaM/CML proteins from passion fruit, *Arabidopsis*, rice, and *Populus trichocarpa* using the neighbor-joining method and the same parameters. The phylogenetic tree was visualized using iTOL (https://itol.embl.de/).

Gene structure, conserved motif, and *cis*-elements analysis.

The exon-intron distributions of the *PeCaM*/*PeCML* genes were obtained from the gene feature file (GFF) of the passion fruit genome. The domain distributions of PeCaM/PeCMLs were analyzed using the NCBI-CDD (https://www.ncbi.nlm.nih.gov/cdd) [41] database. The conserved motifs of the PeCaM/PeCMLs were analyzed using the MEME online program (http://memesuite.org/tools/meme) [46], with the parameters as follows: the maximum number of motifs, 10; other default parameters. The gene structures, domain, and conserved motifs of PeCaM/PeCMLs were displayed using TBtools (v1.120) software [39].

The upstream 2000 bp sequences of the *PeCaM*/*PeCML* were retrieved by TBtools (v1.120) software [39]. The *cis*-regulatory elements in the 2000 bp upstream putative promoter region of *PeCaM*/*PeCML* genes were speculated by the PlantCARE website (http://bioinformatics.psb.ugent.be/webtools/plantcare/html/) [47]. And the results were visualized by TBtools (v1.120) software [39].

Chromosome distribution, gene duplication, and synteny of *PeCaM*/*PeCML* genes.

The genome information of *Arabidopsis* was downloaded from the TAIR (http://www.Arabidopsis.org/). The genomic data of four representative species (*Populus trichocarpa*, *Vitis vinifera*, *Oryza sativa* L., and *Zea mays*) were downloaded from the Ensembl database (http://plants.ensembl.org/index.html).

The chromosome distribution information of *PeCaM*/*PeCML* genes was visualized using Circos [48] in TBtools (v1.120) software according to the GFF file of the passion fruit genome. The gene duplication events of the *PeCaM*/*PeCML* genes were analyzed by MCScanX [49] and graphics were displayed using Advanced Circos and Dual Synteny Plot for MCScanX in TBtools (v1.120) software [39]. Based on screening criteria of > 75% identity and query coverage over 75% gene length, the Ka (non-synonymous substitution) and Ks (synonymous substitution) of *PeCaM*/*PeCML* genes were calculated using Simple Ka/Ks Calculator in TBtools (v1.120) software [39].

Protein-protein interaction network prediction and homology modeling analysis.

All PeCaM/PeCML protein sequences were submitted to the STRING online website (http://string-db.org). Based on *Arabidopsis* homologous proteins, the protein–protein interaction network of PeCaM/PeCML proteins was predicted and constructed using the medium confidence parameter (0.4). Proteins that do not interact with other proteins were removed. The secondary structure of PeCaM/PeCML proteins was predicted using SOPMA secondary structure prediction (https://npsa-prabi.ibcp.fr/cgi-bin/npsa_automat.pl?page=npsa%20_sopma.html). The PDB database (http://www.rcsb.org/) was used to find the most similar homology to the corresponding PeCaM/PeCML protein. Then, the 3D structure of the PeCaM/PeCML proteins was predicted by the Swiss-Model interactive tool (https://swissmodel.expasy.org/interactive/) with default parameters.

Prediction of transcription factor networks.

The Transcription factors prediction and regulatory network analysis as described by Rizwan et al. [50, 51]. The upstream 1000 bp sequences of the *PeCaM*/*PeCML* were extracted by TBtools (v1.120) software [39]. Then the extracted sequences were submitted to the plant transcriptional regulation map (PTRM) [52] and the transcription factor prediction was performed with p-value ≤ 1e^− 6^. Cytoscape software [53] was used to visualize the transcription factor regulatory network.

Expression patterns analysis based on RNA-seq data.

The purple passion fruit (*Passiflora edulis* sim) used for transcriptomic analysis was planted in the orchard of the Institute of Horticulture, Guangxi Academy of Agricultural Sciences. The RNA-sequencing data of the different floral tissues, including two stages of bract tissues (br1 and br2), two stages of corona filament tissues (ca1 and ca8), two stages of the petal (pe1 and pe8), two stages of sepal tissues (se1 and se8), two stages of stigma tissues (sg1 and sg1), three stages of stamen tissues (st1, st8, and st9), and seven stages of ovule tissues (ov2-ov8), were downloaded from China National Gen-Bank (CNGB) (accession number CNP0005768) [54, 55]. Additionally, the RNA-seq data of four different tissues (roots, flower, leaf, and seed) were obtained from China National Center for Bioinformation (CNCB) (accession number CRA003773) [38]. We used ComBat-seq to remove the batch effect [56]. The heatmap was illustrated by pheatmap packages using R software based on log_2_ (FPKM + 0.01).

Additionally, fruit samples were gathered during the fruit juice formation period (53 days after pollination/DAP), fruit juice color transformation period (60 DAP), peel color transformation period (100 DAP), and fruit ripening period (128 DAP). Detailed sample information is also listed in Supplementary Table S9. RNA extraction and Illumina sequencing were performed as previously described [57]. The TPM (Transcripts Per Kilobase Million) value of the *PeCaM*/*PeCML* genes at different developmental stages of passion fruit was calculated. The expression profile of *PeCaM*/*PeCML* genes at different senescence stages was measured by their TPM value, and the heat map was performed by TBtools (v1.120) software [39].

Abiotic stress treatments.

The plants used for stress treatments were grown in a growth chamber under long-day conditions (16 h light/8 h dark) at 27/22°C (day/night) at the Center for Genomics and Biotechnology, Fujian Agriculture and Forestry University. The 3-week-old passion fruit seedlings were treated with cold (8 ± 2 ℃), heat (45 ± 2 ℃), osmotic (200 mM mannitol), and ABA (100 mM ABA) stress, respectively [54, 55]. For cold and heat stress treatment, healthy seedlings in soil were placed in the growth chamber at 8 ℃ or 45 ℃; For osmotic and salt stress, the passion fruit seedlings were first cultured in 1/2 MS liquid medium for 7 days, and then transferred to fresh 1/2 MS liquid medium containing 100 mM ABA or 200 mM mannitol for stress treatment. Plant leaves were collected from at least three independent plants at 0 h, 24 h, and 48 h time points after stress treatment, and untreated plant samples were collected as the control. Then, all collected samples were rapidly frozen with liquid nitrogen and stored at − 80 ℃ for RNA extraction.

RNA extraction and quantitative real-time PCR.

The total RNA was extracted by the Trizol method (Invitrogen, Carlsbad, CA, USA), and the HiScript III Reverse Transcriptase (Vazyme Biotech, Nanjing, China) was used for the reverse-transcription experiment. The qRT-PCR was performed in the Bio-Rad Real-time PCR system (Foster City, CA, USA), using the SYBR Premix Ex Taq II system (TaKaRa Perfect Real Time) with a 20 µL sample volume, and the primers used has been listed in Supplementary Materials Table S10. Each sample was performed three times technically using a replicate, with a total of three biological replicates for each sample. *EF1a* was used as an internal control to normalize the mRNA levels [58, 59]. The fold change of genes was calculated using the 2^−ΔΔCT^ method [59].

## Results

Identification of CaM/CML TFs in passion fruit.

According to the hidden Markov model (HMM) profile of the EF-hand domain, candidate genes with EF-hand domain were preliminarily screened from the genome database of passion fruit. The redundant sequences were removed manually. Subsequently, EF-hand domain was validated by SMART and NCBI-CDD. As a result, a total of 32 *PeCaM*/*PeCML* genes were identified, which included 1 *PeCaM* genes and 31 *PeCML* genes. All of the PeCaM and PeCML were named according to the position of genes on chromosomes. It is noteworthy that PeCML26 is not only highly homologous to PeCaM1 but also closely related to AtCaMs phylogenetically, but it lacks a typical EF-hand domain. Therefore, it was excluded from the *PeCaM* group and defined as the *PeCML* gene.

Then, the biochemical properties of PeCaM and PeCML proteins were predicted using the ExPASy and Cell-PLoc 2.0 (Table 1). As shown in Table 1, PeCaM1 contains four typical EF hand motifs, and PeCMLs contained two to four EF-hands motifs. The lengths of the protein sequences of PeCaM and PeCML ranged from 84 (PeCML16) to 365 (PeCML28) amino acids, and molecular weight (MWs) varied from 9.23 kDa (PeCML16) to 39.72 kDa (PeCML28). Moreover, the theoretical pI of PeCaM/PeCMLs was from 3.95 (PeCML17) to 9.2 (PeCML20). Most of the predicted grand average of hydropathicity (GRAVY) of PeCaM/PeCMLs was negative, and only a few (3/30) were positive, indicating that most of the PeCaM/PeCMLs were hydrophilic proteins and a few were hydrophobic proteins. Most of PeCaM/PeCML proteins were predicted to be located on the cell membrane, but some were also located on cytoplasm, vacuole, nucleus and chloroplast. The diversity of PeCaM/PeCML proteins localization indicates the different functions of these proteins in calcium signal transduction.

**Table 1:** Physiochemical properties of *PeCaM*/*PeCML* genes

Name	Gene ID	Chr*	Genomic position	CDS (bp)	AA* (bp)	EF hands	AI*	GRAVY*	pI*	MW*	SCL*
PeCaM1	P_edulia090022219.g	Chr9	109,983,158–109,984,054	450	149	4	69.4	-0.619	4.11	16847.67	Cell membrane. Cytoplasm.
PeCML1	P_edulia010003464.g	Chr1	181,503,414–181,504,408	447	148	4	81.69	-0.371	4.41	16822.83	Cell membrane.
PeCML2	P_edulia010004424.g	Chr1	190,286,236–190,286,730	495	164	3	86.04	-0.479	4.35	19046.3	Cell membrane.
PeCML3	P_edulia010004568.g	Chr1	192,311,215–192,311,481	267	88	2	82.16	-0.464	6.5	9813.06	Cell membrane. Vacuole.
PeCML4	P_edulia010005389.g	Chr1	201,475,618–201,476,235	618	205	4	80.83	-0.176	5.21	22724.21	Cell membrane.
PeCML5	P_edulia030007681.g	Chr3	3,281,784–3,282,701	450	149	4	90.27	-0.331	4.07	16920.89	Cell membrane. Cytoplasm.
PeCML6	P_edulia030008532.g	Chr3	141,587,083–141,589,319	510	169	4	60.06	-0.908	4.77	19479.74	Cell membrane.
PeCML7	P_edulia030008581.g	Chr3	144,265,869–144,266,348	480	159	4	60.75	-0.784	4.32	18044.8	Cell membrane.
PeCML8	P_edulia040010147.g	Chr4	19,482,749–19,483,315	567	188	4	57.02	-0.621	5.45	21356.27	Cell membrane.
PeCML9	P_edulia040010149.g	Chr4	19,450,336–19,452,869	510	169	4	62.43	-0.899	4.79	19517.82	Cell membrane.
PeCML10	P_edulia050011968.g	Chr5	33,533,144–33,534,741	660	219	2	98.77	0.074	4.9	24248.06	Cell membrane.
PeCML11	P_edulia050012801.g	Chr5	136,008,585–136,009,133	549	182	4	75.55	-0.236	4.67	19857.33	Cell membrane. Vacuole.
PeCML12	P_edulia060013400.g	Chr6	4,211,288–4,211,713	426	141	4	69.15	-0.445	4.47	15985.06	Cell membrane.
PeCML13	P_edulia060013432.g	Chr6	4,486,540–4,486,965	426	141	4	66.38	-0.463	4.47	16003.09	Cell membrane. Vacuole.
PeCML14	P_edulia060013551.g	Chr6	5,321,179–5,322,372	624	207	4	77.29	-0.632	5.33	23992.34	Cell membrane.
PeCML15	P_edulia060013620.g	Chr6	5,767,807–5,768,508	702	233	4	81.55	-0.257	4.79	25959.59	Cell membrane.
PeCML16	P_edulia060013909.g	Chr6	8,304,127–8,304,381	255	84	2	83.81	-0.146	4.34	9225.42	Cell membrane. Nucleus.
PeCML17	P_edulia060014585.g	Chr6	17,689,786–17,690,253	468	155	2	110.06	0.181	3.95	16581.64	Cell membrane.
PeCML18	P_edulia060014586.g	Chr6	17,743,213–17,743,680	468	155	2	106.32	0.157	4.04	16586.66	Cell membrane.
PeCML19	P_edulia060014994.g	Chr6	25,916,464–25,917,659	447	148	4	85.68	-0.423	4.18	16772.69	Cell membrane. Cytoplasm.
PeCML20	P_edulia060015476.g	Chr6	39,922,343–39,922,612	270	89	2	78.99	-0.733	9.2	10186.48	Cell membrane.
PeCML21	P_edulia060015477.g	Chr6	39,892,012–39,892,287	276	91	2	92.09	-0.41	6.82	10127.49	Cell membrane.
PeCML22	P_edulia060015698.g	Chr6	49,904,556–49,905,038	483	160	4	92.69	-0.124	4.33	17720.26	Vacuole.
PeCML23	P_edulia060016150.g	Chr6	112,507,929–112,509,408	453	150	4	83.2	-0.411	4.01	16955.87	Cell membrane. Cytoplasm.
PeCML24	P_edulia060016160.g	Chr6	112,747,710–112,749,237	453	173	4	74.97	-0.561	4.28	19713.96	Cell membrane. Cytoplasm.
PeCML25	P_edulia070017536.g	Chr7	92,703,797–92,706,086	690	229	4	77.07	-0.432	4.58	26417.94	Cell membrane.
PeCML26	P_edulia080018839.g	Chr8	572,635–573,788	426	141	3	70.57	-0.594	4.16	16013.82	Cell membrane. Cytoplasm.
PeCML27	P_edulia080019530.g	Chr8	104,349,920–104,354,634	765	254	2	63.74	-0.539	6.38	28670.86	Cell membrane. Nucleus.
PeCML28	P_edulia080019893.g	Chr8	113,402,328–113,405,389	1098	365	2	89.15	-0.255	4.73	39716.99	Chloroplast. Nucleus.
PeCML29	P_edulia090021448.g	Chr9	12,862,699–12,863,376	678	225	4	87.47	-0.191	4.49	24702.96	Cell membrane. Vacuole.
PeCML30	P_eduliaContig90023069.g	Contig9	12,346–17,050	765	207	2	77.29	-0.635	5.33	23992.34	Cell membrane. Nucleus.
PeCML31	P_eduliaContig200022648.g	Contig20	33,817–35,010	624	254	4	63.74	-0.537	6.16	28595.75	Cell membrane.

Chr*-chromosome NO.; AA*-amino acid/protein length; AI*--aliphatic index; GRAVY*-grand average of hydropathicity; pI*-isoelectric point; MW*-molecular weight (Da); SCL*-Sub-cellular localization.

Phylogenetic analysis of CaMs/CMLs.

**Fig. 1:**
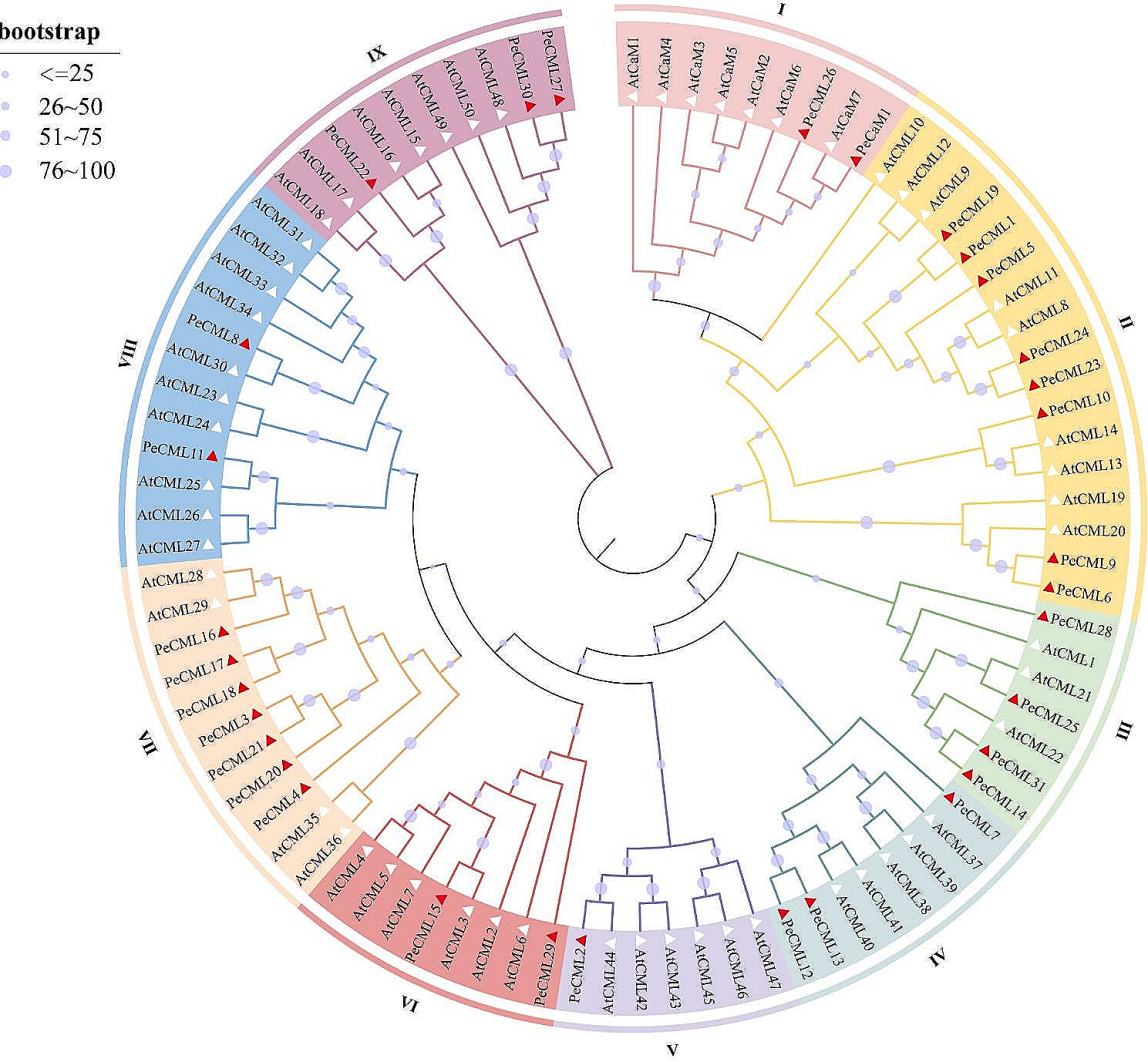
Phylogenetic tree of CaM and CML proteins from passion fruit (Pe) and *Arabidopsis thaliana* (At). The white and red triangles represented genes from *Arabidopsis* and passion fruit, respectively. All 9 groups of CaM/CML were well separated in different clades and represented by different colors

To analyze the phylogenetic relationship and gene function of PeCaMs and PeCMLs, a neighbor-joining (NJ) tree containing 32 PeCaM/PeCMLs and 57 AtCaMs/AtCMLs was constructed by MEGA software (Fig. 1). According to the topological structure of the phylogenetic tree and the classification of AtCaMs/AtCMLs [30], PeCaM/PeCML proteins and AtCaM/AtCML proteins were clustered into nine groups (Groups I–IX), and each group contained different amounts of PeCaM/PeCML proteins. Group II had the largest number of PeCML members (8), followed by Group VII (7) and Group III (4). Group V is the smallest, which contains only one member (PeCML2). Among them, PeCML26 is most closely related to CaMs, suggesting its potential function as CaM. At the same time, we also performed phylogenetic analysis of PeCaM/PeCML with CaM/CML proteins in *Arabidopsis*, rice, and *Populus trichocarpa* (Supplementary figure S1). Phylogenetic analysis showed that CaM/CML proteins from *Arabidopsis*, rice, *Populus trichocarpa* and passion fruit were highly similar, indicating that homologous members have similar functions.

Gene structure, conserved motifs, and *cis*-elements analysis of PeCaM/PeCML.

**Fig. 2:**
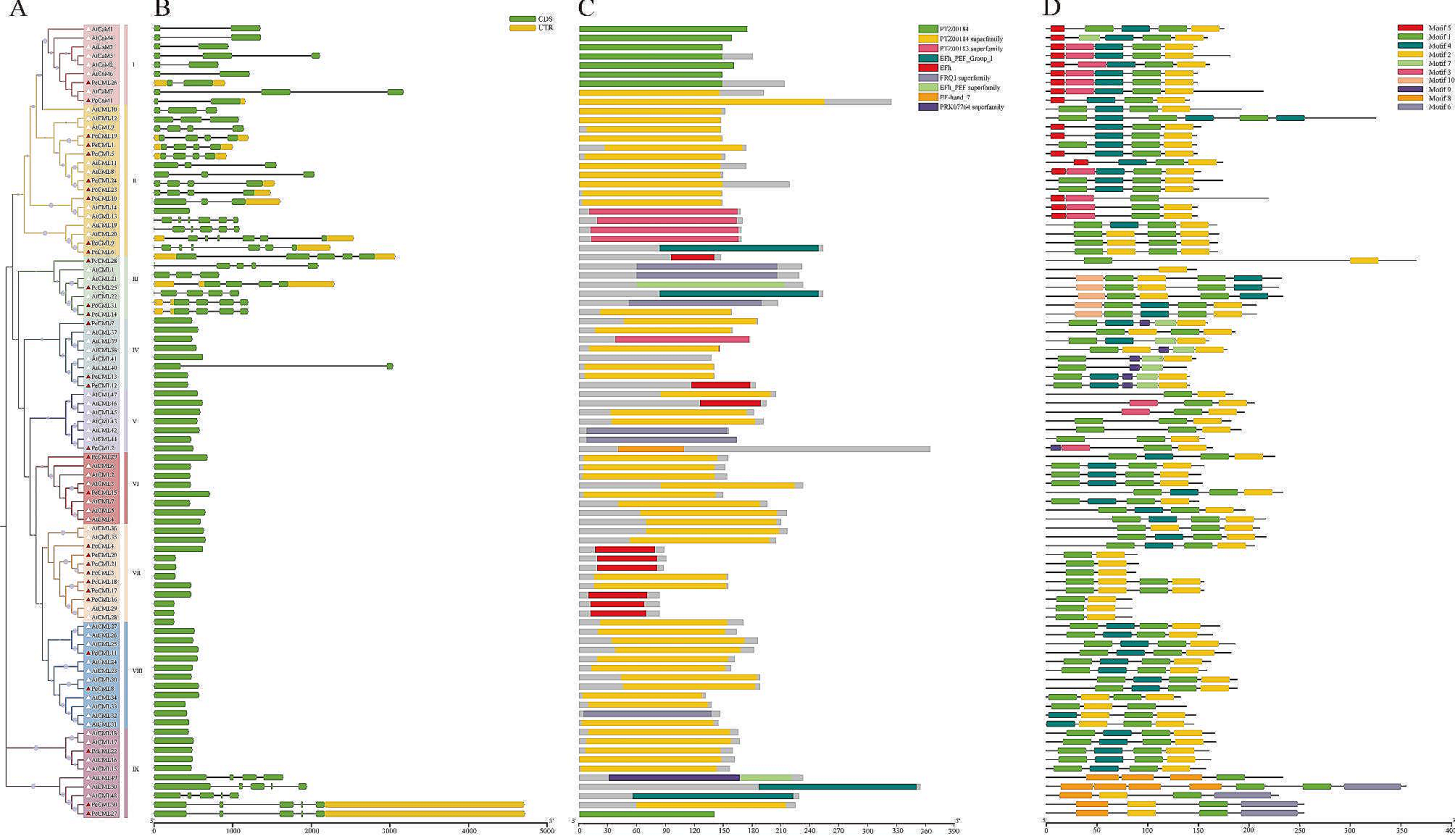
Distributions of gene structure and conserved motifs in CaMs/CMLs. (A) Phylogenetic relationship of PeCaM/PeCMLs and AtCaMs/AtCMLs. (B) Gene structure of PeCaM/PeCMLs and AtCaMs/AtCMLs. (C) The domain distribution of PeCaM/PeCMLs and AtCaMs/AtCMLs. (D) The conserved motifs of PeCaM/PeCMLs and AtCaMs/AtCMLs.

To understand the structural and genetic diversity of *PeCaM*/*PeCML* genes, the gene structure and the composition of conserved motifs of 32 PeCaM/PeCMLs and 57 AtCaMs/AtCMLs proteins were analyzed and displayed according to their phylogenetic relationships (Fig. 2A-D). The exon-intron structure 89 CaMs/CMLs was analyzed and visualized using the TBtools software (Fig. 2B). The result showed that the number of exons varied from 1 to 7 in *CaM*/*CML* genes. Among the 89 CaMs/CMLs, 49 CaMs/CMLs contained a single exon without intron, including 16 PeCMLs. These intronless CaM/CMLs were generally distributed in groups IV to IX. In addition, the genes with similar exon-intron structures, especially in terms of the number of introns, tended to cluster in the same subgroup in the phylogenetic tree. At the same time, we analyzed the domains of PeCaM/PeCMLs and AtCaMs/AtCMLs, and the results showed that most of CMLs belonged to the PTZ00184 superfamily and a few belonged to other superfamilies (Fig. 2C). MEME analysis also proved this similarity (Fig. 2D). The results indicated that motif 1, motif 2, motif 3, and motif 4 represent four different types of EF hands motifs. PeCaM1 contains four types of motifs, which constitute a typical EF-hand domain. At the same time, PeCMLs contain two to four different EF hands motifs. Overall, genes in the same group showed a similar motif composition, however, there were noticeable differences between subgroups suggesting that the motif pattern may be related to the function of the CaM/CML proteins. For instance, motif 6 and motif 8 only appeared in group IX, it may be possible that some genes in the group IX have changed during evolution. The motif analysis of CaMs/CMLs revealed that the members under the same group were highly conserved, further proving the proximity of their evolutionary relationship in the phylogenetic tree.

**Fig. 3:**
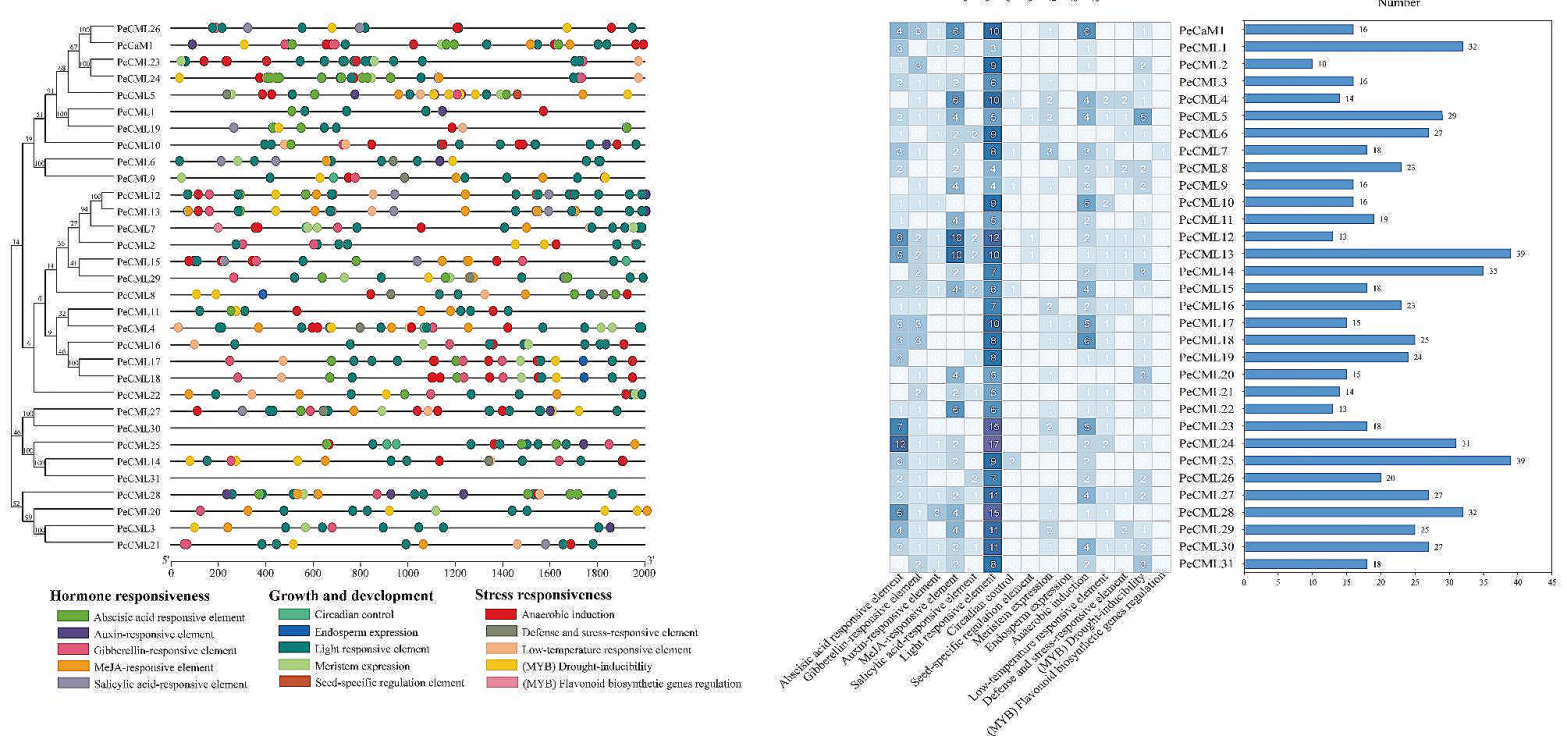
Regulatory elements in the promoter region of *CaM*/*CML* genes in passion fruit. (A) Distribution of *cis*-acting elements identified in the 2000 bp upstream region of *PeCaM*/*PeCMLs* genes. Different colors represent the different types of *cis*-elements. (B) The number of *cis*-acting elements on putative promoters of *PeCaM*/*PeCML* genes

Gene promoters contain many essential *cis*-acting elements that regulate the expression of corresponding genes at the transcriptional level [60]. To explore the transcriptional regulation of PeCaM/PeCMLs, the PlantCARE tool was used to predict the *cis*-elements in 2000 bp upstream sequences of the PeCaM/PeCMLs (Fig. 3). As a result, a total of 15 *cis*-regulatory elements were identified in the promoter, which were divided into three categories: hormone responsiveness, growth and development, stress responsiveness (Supplementary Materials Table S2). Among them, light responsive element was most commonly present in the promoter regions of *PeCaM*/*PeCML* genes, followed by the MeJA-responsive element and ABA-responsive element (ABRE). At the same time, all *PeCaM*/*PeCML* gene promoters contained at least two hormone responsive elements and stress responsive elements, suggesting that *PeCaM*/*PeCMLs* may respond to plant hormones and abiotic stresses. Some elements involved in plant growth and development were observed in a few genes, such as circadian control, endosperm expression, and seed-specific regulation element. These results suggest that the expression of *PeCaM*/*PeCML* genes might be regulated by various *cis*-acting elements related to hormone responsiveness, growth and development, stress responsiveness.

Chromosome localization and collinearity analysis of *PeCaM*/*PeCML* genes.

**Fig. 4:**
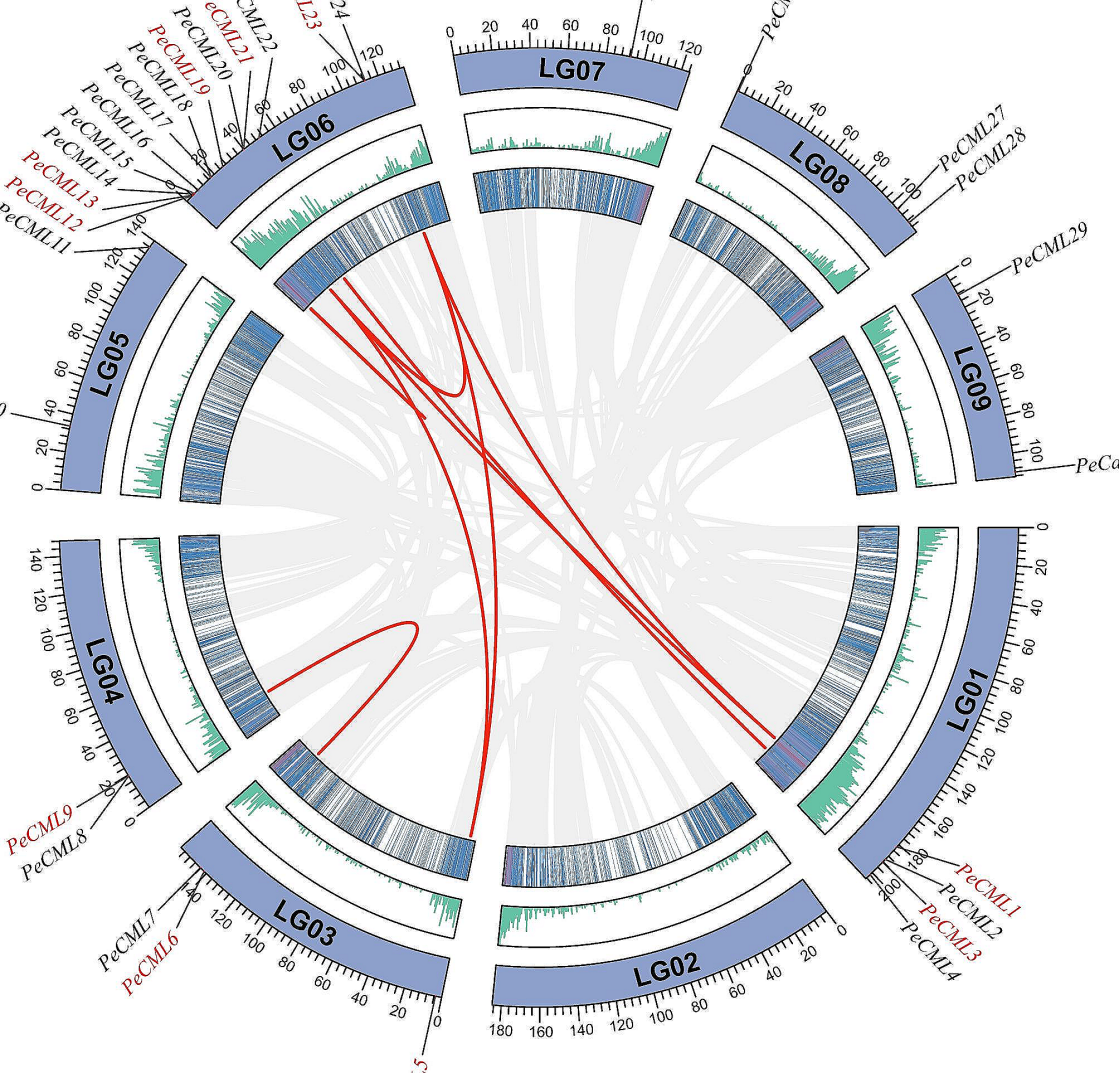
Chromosome location and gene duplication of *PeCaM*/*PeCML* genes on 9 chromosomes. *PeCMLs* marked in red have collinearity, while *PeCMLs* marked in black lack collinearity. The inner rings represent the gene density of each chromosome. The red line represents the segmented and tandem duplication gene pairs among *PeCMLs*.

To study the genetic divergence of the CaM/CML family of passion fruit, the chromosome localization of the *PeCaM*/*PeCML* genes was analyzed (Fig. 4). As a result, the 30 *PeCaM*/*PeCML* genes were unevenly mapped on nine chromosomes, while two were distributed on unassembled scaffolds. Chromosome 6 has the maximum number of *PeCaM*/*PeCML* genes (13 genes, 40.6%), followed by chromosome 1, which contains 4 (12.5%) genes. The remaining chromosomes had between 1 and 3 genes, indicating no correlation between chromosomes and gene number.

The gene duplication event analysis revealed 7 pairs of segmentally duplicated genes and 1 pairs of tandemly duplicated genes were identified in *PeCaM*/*PeCMLs* (Fig. 4, Supplementary Materials Table S3-1). The only tandem duplication event occurred on chromosome 6, whereas segmental duplication events occurred on chromosomes 1, 3, 4, and 6. Of the 32 genes, 8 (25.0%) produced 7 segmental duplication gene pairs suggesting that gene duplication events (segmental duplications) contributed significantly to the diversity and evolution of *PeCaM*/*PeCMLs*. Among the 7 pairs of segmental duplication genes, 6 pairs of duplication genes were distributed in group II, indicating that the *PeCML* genes in group II may have functional redundancy. The ratio of Ka (non-synonymous substitution) and Ks (synonymous substitution) can reflect the selection pressure during organism evolution. Therefore, to explore the role of selection pressure in the evolution of the *CaM*/*CML* gene family, the Ka/Ks ratios of homologous genes were obtained (Supplementary Materials Table S3-2). The Ka/Ks ratios of all *PeCaM*/*PeCML* gene pairs were less than 1, indicating that purification selection played a role in the evolution of *PeCaM*/*PeCML* genes.

**Fig. 5:**
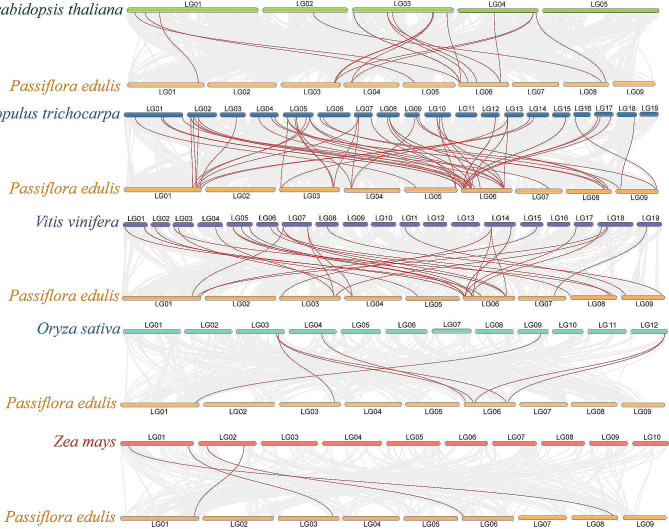
Interspecific collinearity analysis of *PeCaM*/*PeCMLs* and five representative plants (*Arabidopsis thaliana*, *Populus trichocarpa*, *Vitis vinifera*, *Oryza sativa*, and *Zea mays*). Grey lines in the background indicate collinear blocks in passion fruit and other plant genomes, while red lines highlight syntenic *PeCaM*/*PeCML* gene pairs

To further explore the evolutionary mechanisms of *PeCaM*/*PeCML* genes, the synteny and collinearity of passion fruit and five representative plants (*Arabidopsis thaliana*, *Populus trichocarpa*, *Vitis vinifera*, *Oryza sativa*, and *Zea mays*) were analyzed (Fig. 5, Supplementary Materials Table S4). As expected, the genomes of dicotyledon plants *P.trichocarpa* (57 orthologous gene pairs scattered on all chromosomes except chromosome 2), *V.vinifera* (32 orthologous gene pairs scattered on all chromosomes except chromosome 2), and *A.thaliana* (20 orthologous gene pairs scattered on all chromosomes except chromosome 2/7/9) have more collinear gene pairs with the genome of passion fruit, while monocotyledon plants *O.sativa* (7 orthologous gene pairs scattered on chromosomes 1/3/6) and *Z.mays* (4 orthologous gene pairs scattered on chromosomes 1/3/6/8) have less homologous gene pairs. These results show that the divergence of passion fruit occurs after the common ancestor divergence of monocotyledons and dicotyledons. The number of collinear gene pairs between passion fruit and *Populus trichocarpa* was far greater than that among other species, indicating that the genetic relationship between passion fruit and *Populus trichocarpa* is closer than that of the other four species. Among all the *PeCaM*/*PeCMLs*, two genes (*PeCML7* and *PeCML15*) had collinearity with all five species, suggesting that *PeCML7* and *PeCML15* played an important role in the evolution of *CaM*/*CML* gene family.

Protein interactions and homology modeling analysis of PeCaM/PeCML protein.

**Fig. 6:**
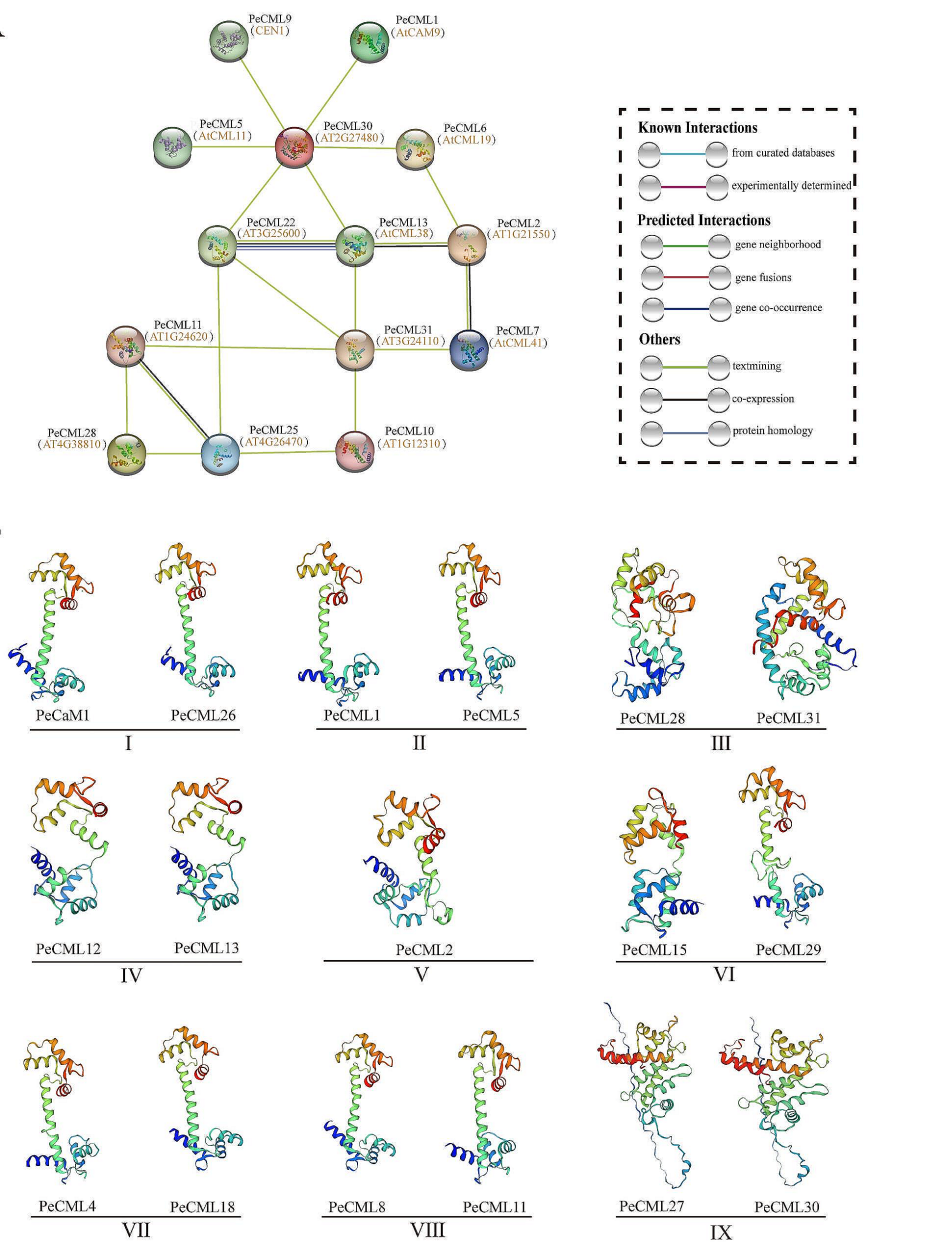
Protein–protein interaction and predicted 3D models of PeCaM/PeCML proteins

In order to better study the biological function and regulatory network of PeCaM/PeCMLs, the protein-protein interaction network was predicted based on known proteins in *Arabidopsis* (Fig. 6). Among them, 26 PeCaM/PeCML proteins are related to known *Arabidopsis* proteins (Supplementary Table S5). The results showed that there were 14 nodes in the PeCaM/PeCMLs protein interaction network, and most of the nodes interacted with multiple other nodes. In addition to PeCML1, PeCML5, and PeCML9 proteins interacting with only one protein, other proteins showed more complex protein interactions, of which PeCML29 interacted with the largest number of proteins. The co-expression of PeCML13 with PeCML2 and PeCML22, and PeCML2 with PeCML7 indicates that they can form a complex biological network to further regulate cell function. The prediction of the protein interaction network showed the interaction between multiple proteins, which indicated the functional diversity of PeCaM/PeCML proteins.

The secondary structure of the protein mainly includes the α-helix, extended strand, β-turn and random coil. We analyzed the secondary structure of all PeCaM/PeCML proteins (Supplementary Table S6-1), and found that the α-helix had the largest proportion in the secondary structure, such as PeCML26 (65.96%); followed by random coil, such as PeCML30 (49.61%); followed by extended strand, such as PeCML10 (12.79%); the proportion of β-turn is the smallest, such as PeCML12 (12.06%). Since each EF-hand motif is composed of α-helix connected by 12 amino acid residues, while PeCaM/PeCML protein contains two to four EF-hand motifs, which may be the reason for the high proportion of α-helix in the secondary structure. In addition, we also predicted the 3D structure of PeCaM/PeCML proteins by SWISS-MODEL database (Supplementary Figure S2). The highest GMQE was selected as the best structure of PeCaM/PeCML proteins (Supplementary Table S6-2). The results showed that most PeCaM/PeCML proteins contain classical EF-loops, which is the core region of the EF-hand domain binding Ca^2+^. PeCaM/PeCML proteins can bind to Ca^2+^ through the EF-loop and enter the activated state, resulting in changes in its conformation, thereby specifically activating the target protein to exert cell function. In addition, some PeCML proteins exhibit different 3D structures, indicating the functional diversity of PeCML proteins.

Prediction of transcription factor regulatory network of *PeCaM*/*PeCML* genes.

**Fig. 7:**
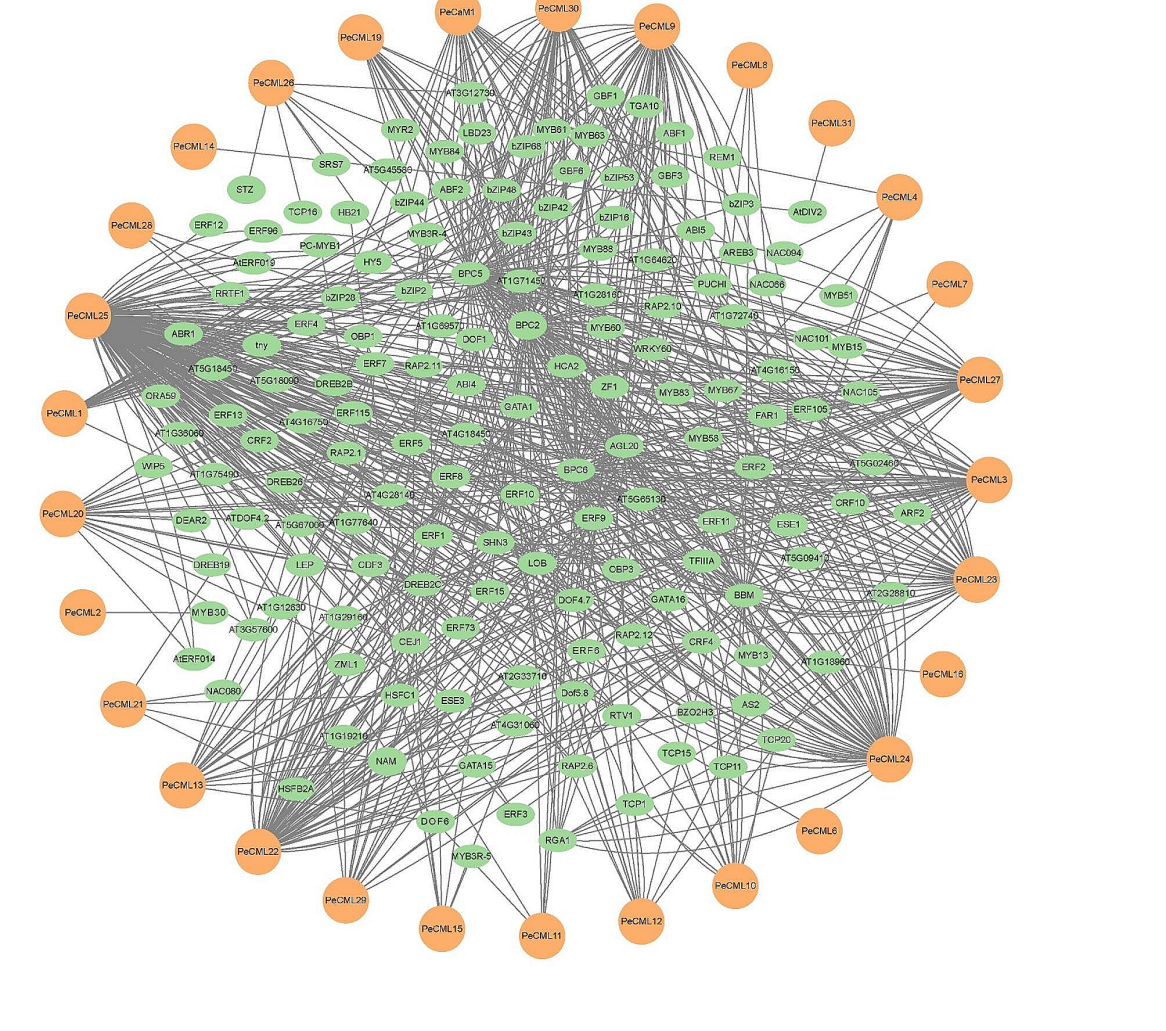
The putative transcription factor regulatory network of *PeCaM*/*PeCML* genes. Green oval nodes represent transcription factors; the orange round nodes represent the *PeCaM*/*PeCML* genes

In order to explore the potential regulatory network of *PeCaM*/*PeCML* gene family, the potential TFs were predicted for 1000 bp upstream of all *PeCaM*/*PeCML* genes, and the TF regulatory network was constructed by Cytoscape. The results showed that the *PeCaM*/*PeCML* genes were regulated by a variety of transcription factor families, including ERF, MYB, bHLH, BBR-BPC, AP2 and bZIP (Fig. 7 and Supplementary Table S7). Among them, ERF family had the most members (260), followed by BBR-BPC (154), Dof (71), MIKC_MADS (42) and bZIP (38). Among all 32 *PeCaM*/*PeCML* genes, *PeCML25* was targeted by the most TFs (186), followed by *PeCML22* (62), *PeCML24* (60), and *PeCML9* (50), while *PeCML2*, *PeCML6*, *PeCML14*, and *PeCML31* were targeted by only one TF. Different *PeCaM*/*PeCML* genes were targeted by diverse types and numbers of TF families. For example, the families targeting *PeCML25* include ERF (126), Dof (26) and MIKC_MADS (15); the families targeting *PeCML23* gene were BBR-BPC (35) and AP2 (3). Among the TFs targeting *PeCaM*/*PeCML* genes, there were many TFs related to plant growth and development and response to biological and abiotic stress, such as ERF, bZIP, BBR-BPC, WRKY, bHLH, and AP2.

Expression analysis of *PeCaM*/*PeCML* genes based on transcriptomic data.

**Fig. 8:**
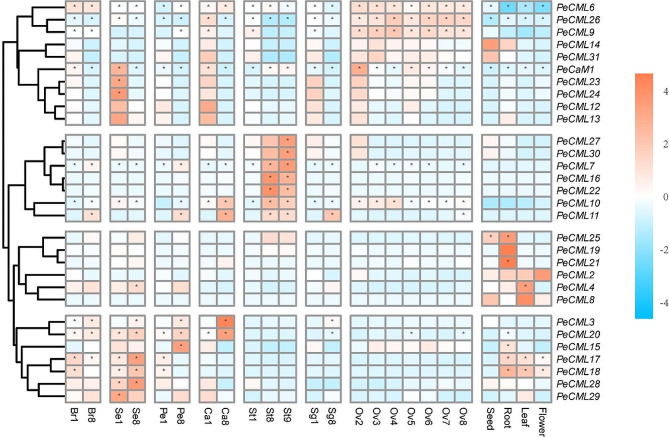
The expressional pattern of *PeCaM*/*PeCML* genes in passion fruit. The expression of *PeCaM*/*PeCMLs* in diverse tissues at different developmental stages (bract (br); sepal (se); petal (pe); corona filament (ca.); stamen (st); stigma (sg); ovule (ov); numbers represent developmental stages, 1 and 2 was early stage, 8 was the later stage. Blue or orange colors represent the difference in expression levels, respectively. The heatmap was created according to the log_2_(FPKM + 0.01) value of *PeCaM*/*PeCMLs* and normalized by row. The FPKM value higher than 50 was shown as abundant genes, and marked with “*”

To explore the possible functions of the *PeCaM*/*PeCMLs*, the expression profiles of the 32 *PeCaM*/*PeCMLs* in the various tissues at different developmental stages were characterized using RNA-seq data (Fig. 8). Genes with low expression levels in all samples were filtered out. The results showed the expression variation of *PeCaM*/*PeCML* genes at different stages of flower development. For example, *PeCML6*, *PeCML9*, and *PeCML26* were highly expressed throughout the ovule development stage. *PeCML12*, *PeCML13*, *PeCML23*, and *PeCML24* were highly expressed in the early stages of sepal, corona filament, stigma and stamen development. *PeCaM1* was highly expressed in all vegetative tissues and various developmental stages of floral tissues, with higher expression patterns in sepal and ovule development stages, indicating that *PeCaM1* is ubiquitous in the growth and development of passion fruit and involved in early flower development. In addition, seven genes (*PeCML7*, *PeCML10*, *PeCML11*, *PeCML16*, *PeCML22*, *PeCML27*, and *PeCML30*) showed high expression levels in the late stage of stamen development, suggesting that these genes may play essential roles in stamen development. Furthermore, we also found that some *PeCML* genes were specifically expressed in specific tissues. For example, *PeCML19* and *PeCML21* were highly expressed in roots. Interestingly, some *PeCML* genes were preferentially expressed in multiple vegetative tissues, such as *PeCML17* and *PeCML18*, which were highly expressed in root, leaf, and flower tissues. These results indicated that *PeCaM*/*PeCML* genes exhibited a certain degree of tissue expression specificity.

**Fig. 9:**
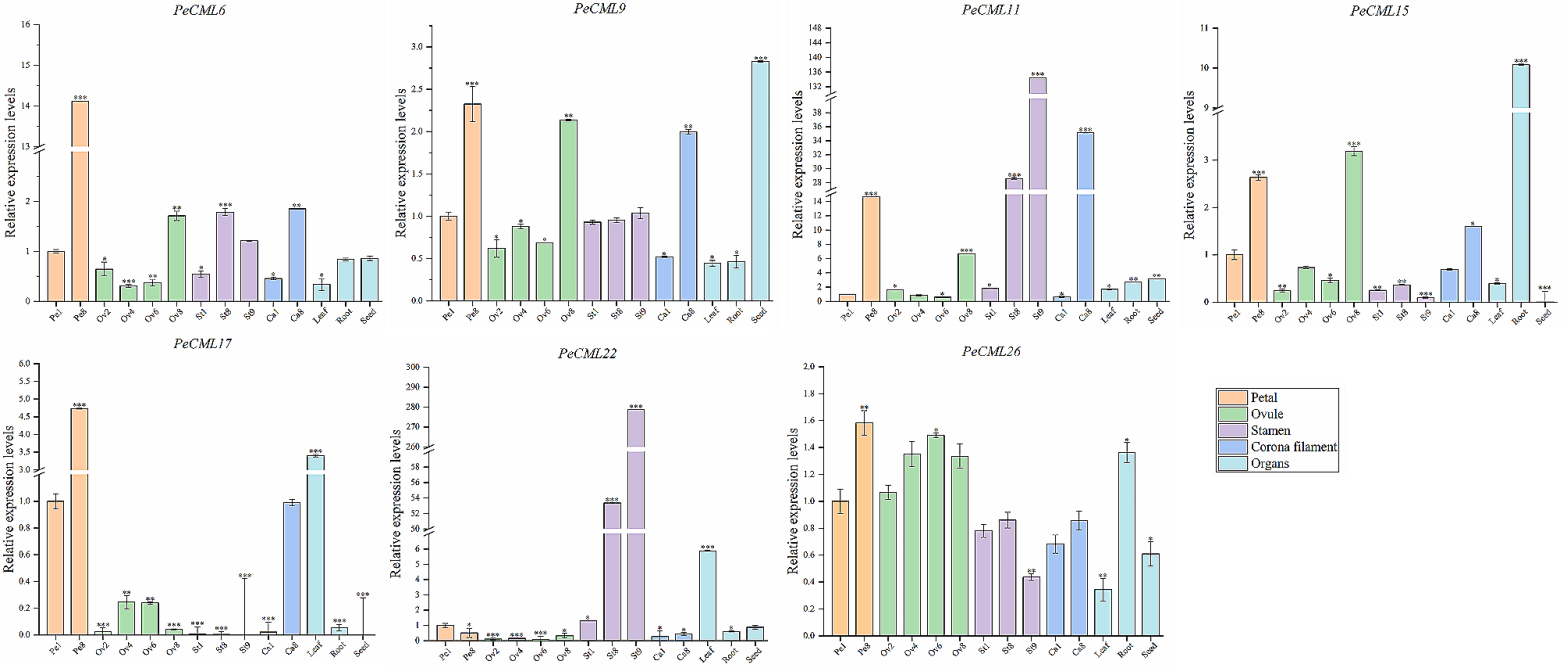
qRT-PCR analysis of 7 genes (*PeCML6*, *PeCML9*, *PeCML11*, *PeCML15*, *PeCML17*, *PeCML22* and *PeCML26*) in 4 floral tissues (petal, ovule, corona filament, and stamen) and three organs. All experiments were repeated three times. The error bar represents the standard deviation (SD) of three replicates. Asterisks indicate significant differences in transcript levels compared with those of early stage of petal development (pe1). (*P < 0.05, **P < 0.01, ***P < 0.001)

In order to verify the expression of *PeCaM*/*PeCML* genes in floral tissues development and various organs, qRT-PCR was used to study the expression profiles of seven representative *PeCML* genes (*PeCML6*, *PeCML9*, *PeCML11*, *PeCML15*, *PeCML17*, *PeCML22* and *PeCML26*) in the same flower and organ sample (Fig. 9). Overall, the trends of the qRT-PCRs for the six *PeCML* genes were consistent with the results of RNA-seq analysis. *PeCML11* was highly expressed at the late developmental stages in multiple floral tissues such as petals, ovules, stamens and corona filament, and *PeCML22* was significantly highly expressed during late stamen development. *PeCML15* was highly expressed in late developmental stages of petals and ovules and specifically expressed in root organ. In addition, *PeCML6* and *PeCML9* were highly expressed in the late developmental stages of petals and ovules.

**Fig. 10:**
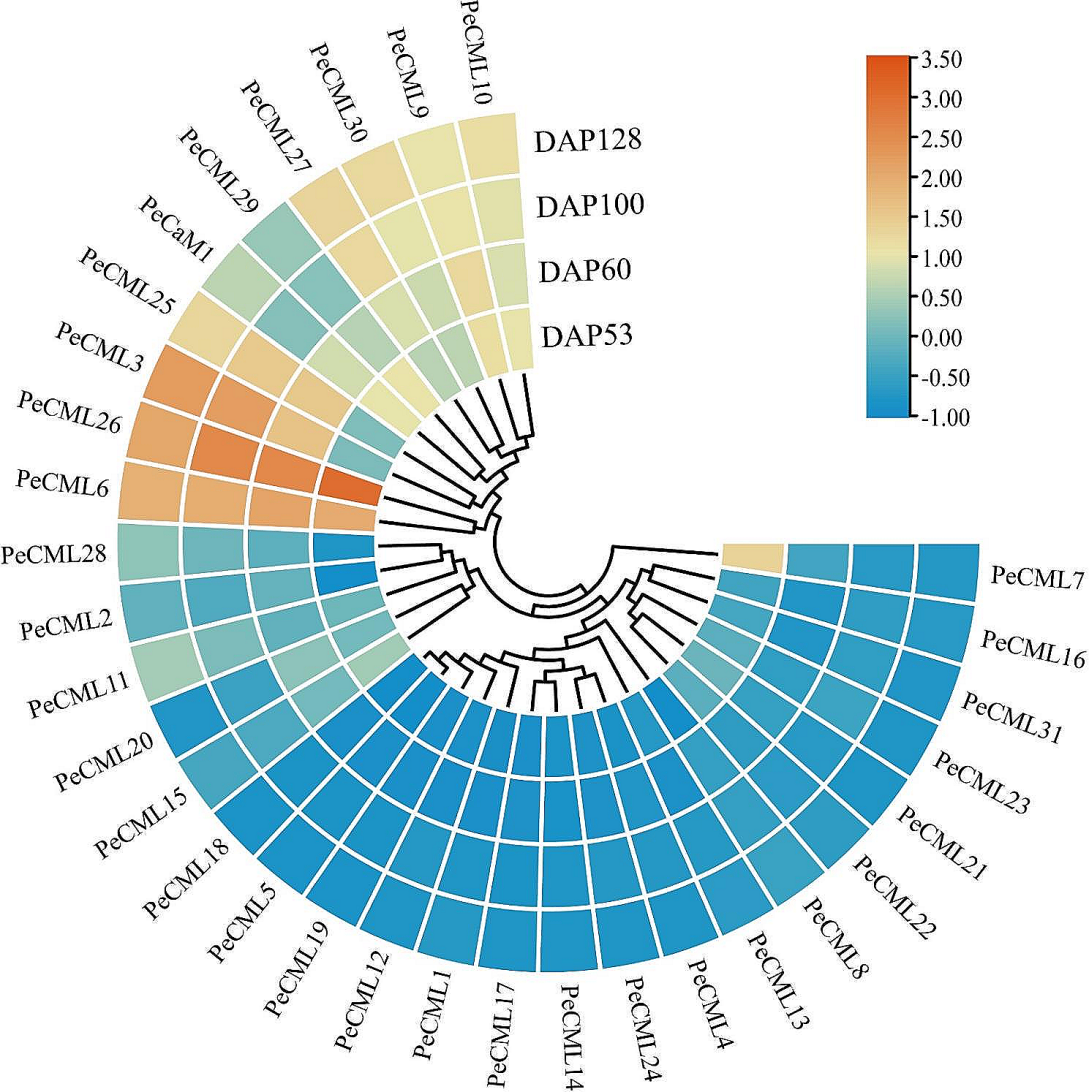
The expression profiles of *PeCaM*/*PeCML* genes at different stages of passion fruit. DAP, the day after pollination. Blue or orange colors represent the difference in expression levels, respectively

We also analyzed the expression patterns of *PeCaM*/*PeCML* genes at different developmental stages after the pollination based on RNA-seq (Fig. 10). Among these *PeCaM*/*PeCML* genes, the expression levels of *PeCML3*, *PeCML27*, and *PeCML30* were continuously increased with the prolongation of the time after pollination, while the expression levels of *PeCML15* were decreased. In addition, other *PeCML* genes such as *PeCML6* and *PeCML26* exhibited fluctuating expression patterns. Interestingly, compared to other *PeCML* genes, *PeCML26* had extremely high expression levels during fruit development, suggesting that *PeCML26* may be involved in the fruit development of passion fruit. Besides, we verified the expression level of *PeCML26* at different stages of passion fruit by qRT-PCR, and the result was consistent with RNA-seq results (Supplementary figure S3). These results indicate that different *PeCMLs* play vital roles in various stages of passion fruit development.

Expression pattern of *PeCaM*/*PeCML* genes in response to abiotic stress.

**Fig. 11:**
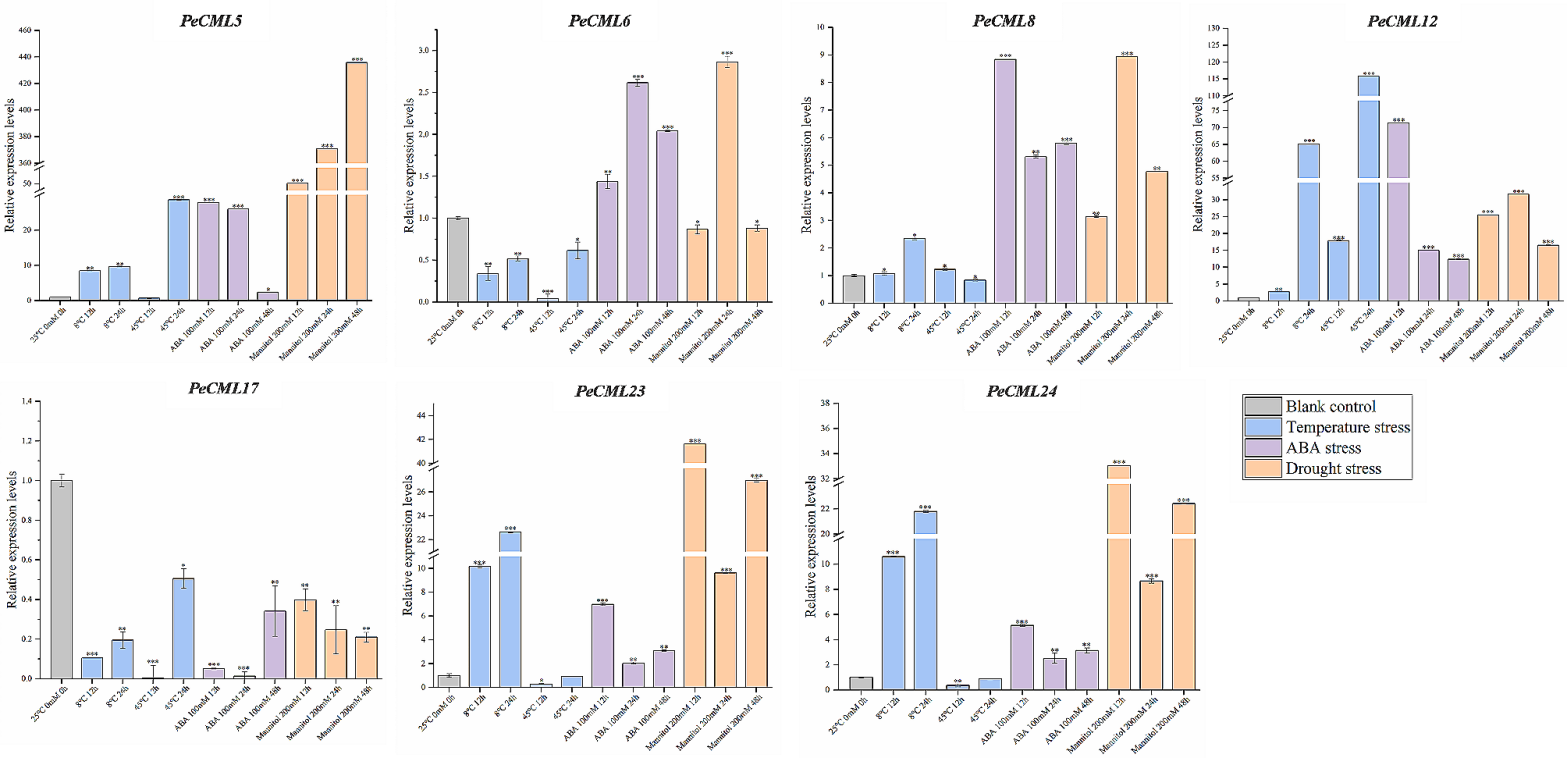
Expression patterns of seven *PeCML* genes under cold, heat, osmotic, and ABA stress. The ordinate is the relative expression level (multiple) of *PeCMLs* compared with the internal reference gene (*EF1a*). All experiments were repeated three times. The error bar represents the standard deviation (SD). Asterisks indicate significant differences in transcript levels compared to control values (*P < 0.05, **P < 0.01, ***P < 0.001)

In order to explore whether the *PeCaM*/*PeCML* genes are involved in the response of passion fruit to abiotic stress, seven *PeCML* genes (*PeCML5*, *PeCML6*, *PeCML8*, *PeCML12*, *PeCML17*, *PeCML23*, and *PeCML24*) were randomly selected to analyze their expression patterns under temperature stress, ABA stress and osmotic stress using qRT-PCR methods (Fig. 11). Under low-temperature treatment, five genes (*PeCML5*, *PeCML8*, *PeCML12*, *PeCML23*, and *PeCML24*)) had similar expression patterns and showed a continuous increase with the treatment time. The relative expression level of *PeCML6* and *PeCML17* first decreased and then increased with the treatment time. Whereas under heat treatment, only the expression levels of *PeCML12* significantly up-regulated, and other *PeCML* genes (*PeCML5*, *PeCML6*, *PeCML8*, *PeCML17*, *PeCML23*, and *PeCML24*) showed fluctuated expression patterns. In terms of ABA stress, all the seven genes showed a fluctuating expression pattern, but the expression levels of the other six genes except *PeCML17* were increased compared with the control at different times, while the expression level of *PeCML17* was decreased compared with the control. During the osmotic treatment, *PeCML6*, *PeCML8*, *PeCML12*, *PeCML23*, and *PeCML24* exhibited fluctuated expression patterns, the expression level of *PeCML5* was significantly up-regulated and the expression level of *PeCML17* was down-regulated. Interestingly, *PeCML5* exhibited significantly up-regulated expression levels in response to all the stresses. The expression level of *PeCML12* showed an increasing trend only under cold and heat treatments, while *PeCML23* and *PeCML24* increased under only cold stress conditions.

**Fig. 12:**
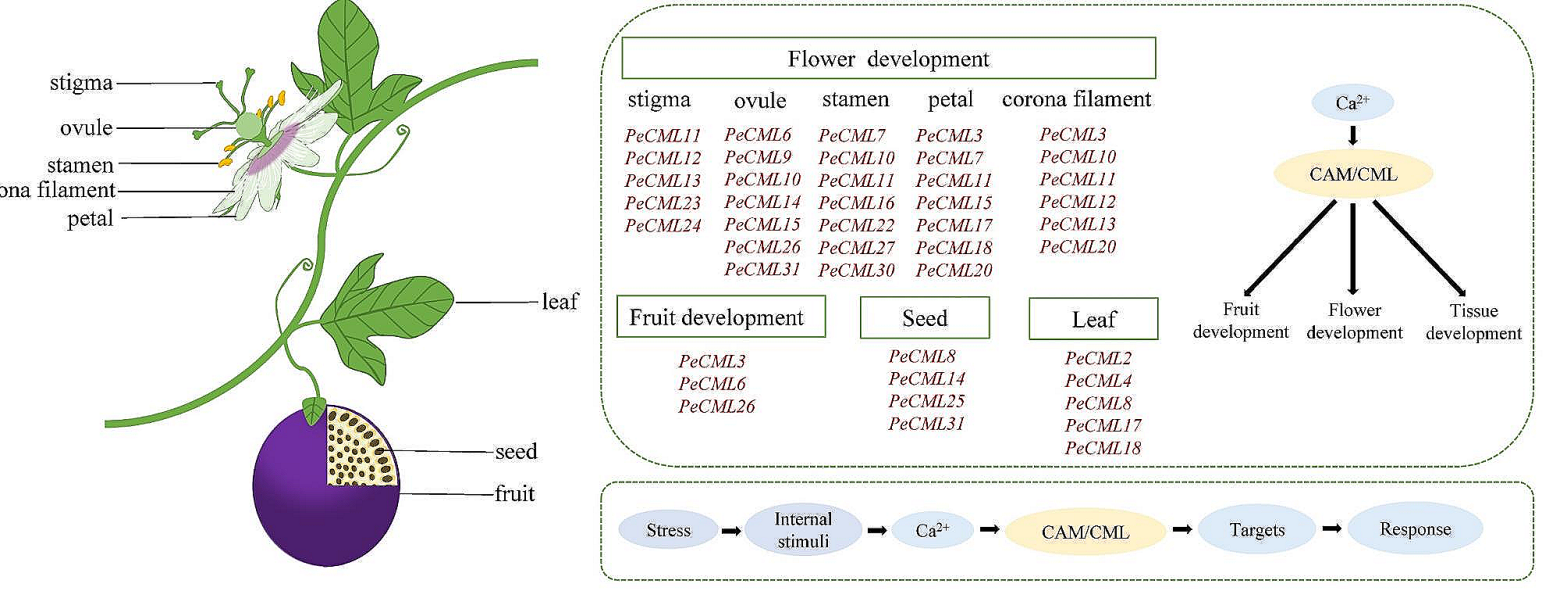
The potential function of *CaM*/*CML* genes in passion fruit. The schematic diagram model of *PeCaM*/*PeCML* gene expression patterns during flower and fruit development and its application. The *PeCML* genes marked in red were highly expressed in the corresponding tissue

## Discussion

Increasing investigations suggests that Ca^2+^ is a second messenger involved in plant growth and development and in response to various biotic and abiotic signals [1, 2, 61]. Plants have evolved a variety of Ca^2+^ sensors that bind to calcium ions. Calmodulin (CaM) and Calmodulin-like (CML) proteins are the main calcium sensors in eukaryotes. They have been extensively studied in a variety of plants, such as *Arabidopsis* [30], rice [31], apple [34], grape [32]. Passion fruit is a famous and economically valuable fruit crop whose growth and development processes are susceptible to various environmental factor changes [62]. Although many *PeCaM*/*PeCML* genes have been reported in numerous plants, limit information is available about the passion fruit *PeCaM*/*PeCML* genes. For the first time, we systematically studied the *CaM*/*CML* gene family of passion fruit with bioinformatics tools and expression profiles.

In this study, a total of 32 *PeCaM*/*PeCML* genes were identified in the passion fruit genome, including 1 *PeCaM* gene and 31 *PeCML* genes. Compared with other plants, the number of *PeCMLs* was more than *Ginkgo biloba* (21) [63] and Chinese jujube (23) [64] but was less than that in *Arabidopsis* (50) [30], rice (32) [31], grape (62) [32] and apple (58) [34]. Notably, the number of *PeCMLs* genes was not significantly correlated with genome size. For example, *Arabidopsis* has 50 *CML* gene family members with a 125 Mb genome size, while the genome size of passion fruit is more than 9 times that of *Arabidopsis* with fewer number of *PeCMLs*. The number of *PeCMLs* in grape was about twice to that of in passion fruit. In the process of evolution, grape experienced three genome-wide replication events while passion fruit experienced only twice [38, 65]. This indicates that the number of *PeCML* genes in plants is not related to genome size, but closely related to the evolution of species. According to the phylogenetic analysis results, all the *CaM*/*CML* genes from passion fruit (32 *PeCaM*/*PeCMLs*) and *Arabidopsis* (57 *AtCaM*/*AtCMLs*) were divided into 9 groups. All groups contained the CaM/CML members from both passion fruit and *Arabidopsis*, suggesting that they might be derived from a common ancestor. In addition, the neighbor-joining phylogenetic tree constructed with *Arabidopsis*, rice, and *P. trichocarpa* CaM/CMLs showed their evolutionary relationships and potential functional similarities (Supplementary Fig. 1).

The members clustered in the same subgroup share similar exon-intron structure, motif composition, and distribution order. Previous studies have shown that most *CML* genes have no intron, while some genes have less than ten introns [30, 31, 66]. As previously other plants, most of the *PeCaM*/*PeCML* genes were intron-less, and other genes had no more than 6 introns, which could indicate their conserved functions in different species. Motif 1, motif 2, motif 3, and motif 4 were highly conserved in most PeCaM/PeCML proteins and represented different types of EF hands motifs, which were critical for the functional specificities of these TFs [66]. The composition of the EF-hand motif also reveals that PeCaM/PeCMLs has a certain degree of diversity. Most of them (20 out of 32) have four different or identical EF-hand motifs, and the remaining members have at least one EF-hand motif. The number of EF-hand motifs of *PeCaM*/*PeCML* genes was consistent in the same group. In contrast, the structure of different groups varied considerably. For instance, group III and group IV have considerably different motif compositions from other subgroups, and motif 10 is unique to group III, suggesting that the of *PeCML* genes in group III may have undergone distinct evolutionary development and have specific functions. The expression profiles of *PeCML* genes in group III were different. For example, *PeCML25* was highly expressed in root and seed tissues, while *PeCML28* was highly expressed in sepals. Thus, the structural diversity among different subgroups might contribute to the functional diversity of the CaM/CML gene family.

Gene replication events play an important role in the expansion of gene family members, the segmental duplication and tandem duplication are the main reasons for the expansion of gene families in the genome [67]. Here, in the passion fruit *PeCaM*/*PeCML* gene family, 1 pairs of tandem duplication and 7 pairs of segmental duplication genes were identified, indicating that the segmental duplication events were the main source for the expansion of the *CaM*/*CML* gene family of passion fruit. The Ka/Ks value of all duplicated gene pairs are less than 1, indicating that *PeCaM*/*PeCML* genes have gone through purifying selection during evolution. Some duplicated genes showed similar expression patterns and clustered in the same subfamily, such as *PeCML6* and *PeCML9* from the group II were both highly expressed throughout the ovule development stage, indicating that they might have functional redundancy. However, most duplicated gene pairs exhibited diverse expression patterns, which indicates that the gene members of the CaM/CML family of passion fruit may have functional differentiation. For example, although *PeCML19* and *PeCML23* were in group II, they showed completely different expression patterns. *PeCML19* showed low expression levels at different developmental stages of various floral tissues, while *PeCML23* was highly expressed in the early developmental stages of the sepal, corona filament, stigma, and ovule. The difference in the expression patterns of these duplicate genes infers the functional diversity of *PeCaM*/*PeCML* genes. According to the collinearity analysis, the collinearity relationship between passion fruit and dicotyledon plants is much closer than that between passion fruit and monocotyledon plants. Among them, the number of homologous genes between passion fruit and *Populus trichocarpa* was the maximum, indicating that these homologous genes may have the same ancestors and retain the corresponding functions.

Interaction network analysis can better understand the biological functions and molecular pathways of proteins. In this study, protein-protein interaction analysis showed that most PeCaM/PeCML proteins were homologous and interacted with known *Arabidopsis* proteins, indicating that PeCaM/PeCML may have similar functions to the corresponding proteins. Homologous modeling results also showed that gene members from the same group shared similar protein secondary structure and 3D structure features, while PeCaM/PeCML proteins from different groups present different 3D structure features, which supports the classification results. For example, PeCML23 and PeCML24 in II group not only have the same 3D structure but also are highly expressed in the early stage of flower tissue. In summary, the diversity of PeCaM/PeCML protein structure also indicates the difference between protein functions. In addition, we also predicted the TFs in the promoter region of the *PeCaM*/*PeCML* genes and constructed a transcription factor regulatory network. The results showed that ERF, BR-BPC, Dof, MIKC_MADS, bZIP, and AP2 families accounted for the largest proportion. BBR-BPC1 is involved in the regulation of *Arabidopsis* ovule development by regulating SEEDSTICK (STK) [68]. The bZIP protein plays an important role in various biological functions such as plant growth and development, seed maturation, response to light signals and environmental stress [69, 70]. It is worth noting that PeCaM/PeCML proteins that interact with these transcription factors may have similar functions. *PeCaM1* showed high expression levels in various developmental stages of floral tissues and various vegetative tissues. At the same time, it mainly interacted with various transcription factors of bZIP and BBR-BPC families, indicating that *PeCaM1* was mainly involved in plant development through these transcription factors. *PeCML26* also has a similar expression pattern with *PeCaM1* and interacts with multiple BBR-BPC TFs, further illustrating the importance of *PeCML26* in the growth and development of passion fruit. Moreover, *PeCML26* was highly expressed during ovule development and fruit development, further indicating that *PeCML26* plays a crucial role in the development of flowers and fruits in passion fruit. Through qRT-PCR expression analysis, it was found that the expression levels of *PeCML* genes were positively or negatively regulated under various stress conditions, and it interacted with a large number of TFs (Fig. 11). Consistent with the predicted function, these results indicate that PeCaMs/PeCMLs play important roles in various growth and development processes of plants and in response to various biotic and abiotic stresses.

As a popular fruit and ornamental crop, it is of great significance to exploring the regulatory mechanism of flower and fruit development of passion fruit. The biological functions of most PeCaM/PeCMLs remain unclear, and the identification of putative orthologs in different species will provide valuable reference for the functional study of these genes. Previous studies suggested that *AtCML24* and *AtCML25* were involved in the regulation of pollen germination and pollen tube growth in *Arabidopsis* [14, 71]. *PeCML11* clustered with *AtCML24* and *AtCML25* showed a high expression pattern at the late developmental stage of stamen and stigma, suggesting that *PeCML11* may have similar functions in the regulation of pollen germination and pollen tube growth. *PtCML20* is involved in the development of leaves in *Populus trichocarpa* [44]. *PeCML8* clustered in the same subgroup was highly expressed in leaf, suggesting that *PeCML8* may be involved in the leaf development of passion fruit. *PtCML23* shows a gradually upregulated expression trend with flower development [44], and the expression level of *PeCML7* clustered with *PtCML23* increases with flower development, indicating that *PeCML7* may be involved in flower development. *AtCML42* is involved in trichome formation and is broadly expressed in plant tissues (such as flowers, leaves and roots) [72], the homologous *PeCML2* expressed highly in flower, leave and root tissues, suggesting that *PeCML2* may play similar roles in the development of passion fruit tissues. In addition, the tissue expression pattern indicated that *PeCMLs* played an important role in the reproductive development of passion fruit, as shown in Fig. 12. This also provides an important basis for understanding the role of *CML* genes in regulating the development of passion fruit flowers and fruits.

The growth and development of passion fruit are susceptible to the alterations of environmental factors including light, temperature and water [73]. *CaM*/*CML* genes have also been proven to be related to the abiotic stress response [12]. For example, ectopic expression of soybean *GsCML27* in *Arabidopsis* enhances the tolerance of plants to bicarbonate stress [74]. The expression of seven *PeCML* genes was analyzed by qRT-PCR under four abiotic stress conditions, including cold, heat, ABA, and osmotic stress. *OsCML8* showed high expression levels under osmotic and salt stress in rice [75]. *PeCML6* and *OsCML8* were clustered in the same group, and the expression level of *PeCML6* was upregulated under osmotic and salt stress, indicating that *PeCML6* may be involved in Ca^2+^-mediated responses to stimuli. Studies have shown that *AtCML9* is involved in response to cold, ABA and drought treatments [76]. *PeCML5* in the same group as *AtCML9* responded to various stresses. At the same time, *PeCML5* also has the most five MYB binding sites involved in drought inducibility, and its expression was significantly increased under drought stress, indicating that *PeCML5* plays a crucial role in the response of passion fruit to drought stress. *PeCML23* and *PeCML24* contain the most ABA responsive elements and some low-temperature responsive element, and they are highly sensitive to cold and drought stress, indicating their potential functions in the process of cold and drought. These results indicate that the results of *PeCML cis*-elements are consistent with the results of qRT-PCR expression analysis, and *PeCML* genes containing more stress-related *cis*-elements not only respond to multiple stresses but also their expression levels are highly up-regulated. The complex interactions with multiple hormone regulations might enable these *PeCML*s to be involved in diverse biological processes. The response of *PeCMLs* to abiotic stress can alleviate the further damage of abiotic stress to the normal growth of passion fruit, which plays an important role in the development of passion fruit.

## Conclusions

Here, a total of 32 *PeCaM*/*PeCML* genes were identified in the passion fruit (*Passiflora edulis*) genome, including 1 *PeCaM* gene and 31 *PeCML* genes. Based on the phylogenetic relationships, 32 *PeCaM*/*PeCML* genes were divided into 9 groups. The *PeCaM*/*PeCML* genes were randomly distributed on 9 passion fruit chromosomes. Gene structure and motif composition analysis showed that PeCaM/PeCMLs in the same subgroup were highly conserved. Collinearity analysis showed that segmental duplication events contributed to the expansion of the CaM/CML family of passion fruit. *Cis*-regulatory elements of promoters were involved in growth and development, Hormone responsiveness, and stress responsiveness. The interactions among PeCaM/PeCML proteins or with other transcription factors may make the regulatory network of PeCaM/PeCMLs more diverse and complex. The *PeCaM*/*PeCML* genes showed differential expression patterns in floral tissues at different development stages. It is worth noting that PeCML26, which is highly homologous to AtCaM2, not only interacts with multiple BBR-BPC TFs, but also has high expression levels during ovule and fruit development, suggesting that *PeCML26* has potential functions involved in the development of passion fruit flowers and fruits. Additionally, seven *PeCML* genes also showed a differential response under various abiotic stresses (cold, heat, ABA, and drought). This research lays the foundation for future investigations and validation of the potential function of *PeCaM*/*PeCML* genes in the growth and development of passion fruit.

### Electronic supplementary material

Below is the link to the electronic supplementary material.

Supplementary Material 1

Supplementary Material 2

Supplementary Material 3

Supplementary Material 4

Supplementary Material 5

Supplementary Material 6

Supplementary Material 7

Supplementary Material 8

Supplementary Material 9

Supplementary Material 10

Supplementary Material 11

Supplementary Material 12

Supplementary Material 13

## Data Availability

The data presented in this study are available in the article, Supplementary Materials, and online repositories. RNA-seq data used in this work were deposited in China National GenBank (CNGB) under (accession number CNP0005768 and CNP0005717) and China National Center for Bioinformation (CNCB) (accession number CRA003773).

## Abbreviations

ABA: Abscisic acid
CaM: Calmodulin
CML: Calmodulin-like
FPKM: Fragments Per Kilobase of exon model per Million mapped fragments
Gravy: Grand average of hydropathicity
HMM: Hidden markov mode
MEME: Multiple EM for motif elicitation
qRT-PCR: Quantitative real-time PCR
