# Supplementary material for: Genome-wide identification and expression analysis of calmodulin and calmodulin-like genes in passion fruit (Passiflora edulis) and their involvement in flower and fruit development
Source: BMC Plant Biol. 2024 Jul 3;24:626. doi: 10.1186/s12870-024-05295-y (PMC11220982; doi:10.1186/s12870-024-05295-y)
Supplement: Supplementary file 10 — Supplementary Material 10 [file 12870_2024_5295_MOESM10_ESM.pdf]

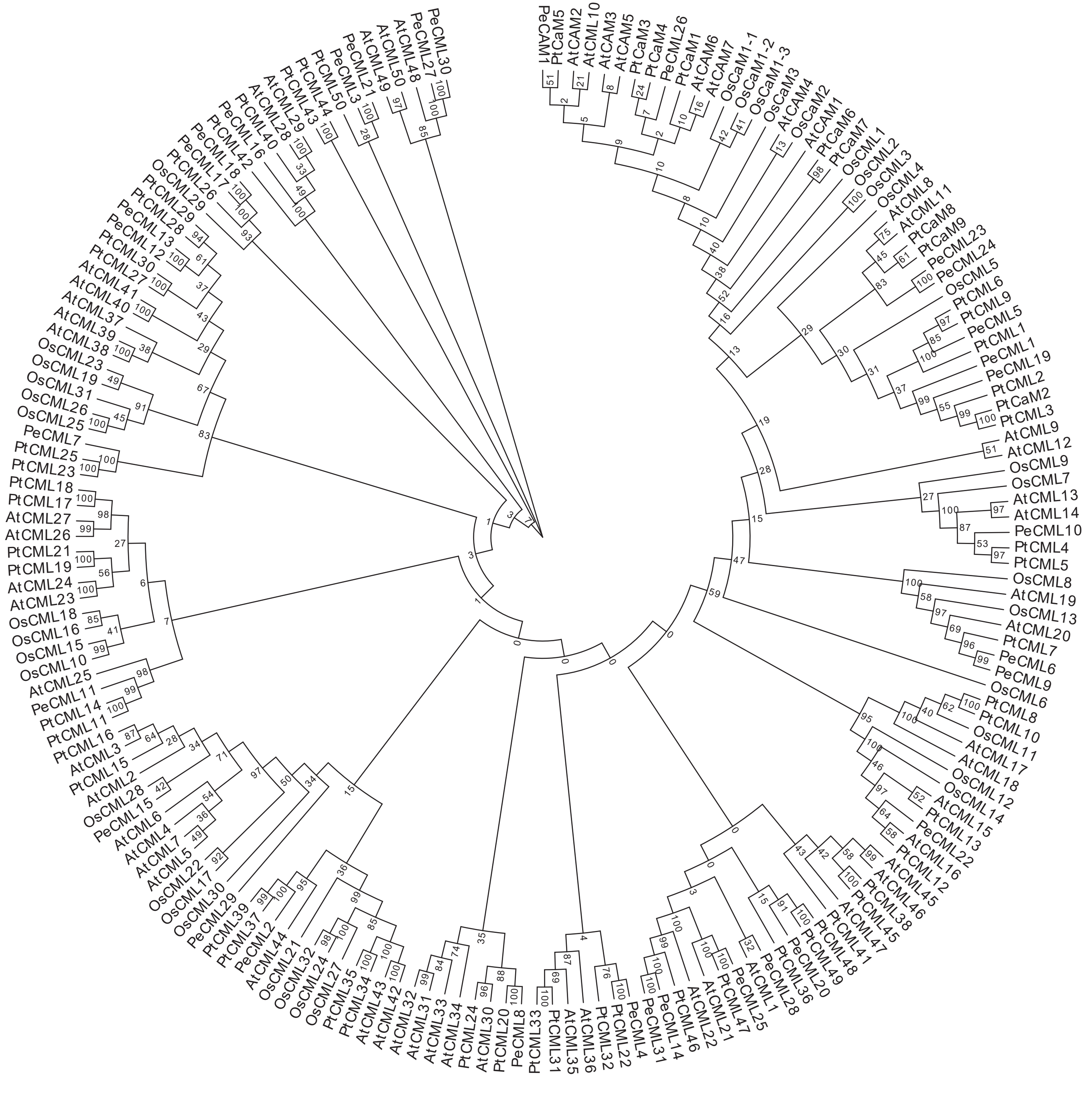

Supplementary Materials Figure S1: Phylogenetic tree of CaM and CML proteins from passion fruit, *Arabidopsis*, rice, and *Populus trichocarpa*.
