# Supplementary material for: Genome-wide identification and expression analysis of calmodulin and calmodulin-like genes in passion fruit (Passiflora edulis) and their involvement in flower and fruit development
Source: BMC Plant Biol. 2024 Jul 3;24:626. doi: 10.1186/s12870-024-05295-y (PMC11220982; doi:10.1186/s12870-024-05295-y)
Supplement: Supplementary file 12 — Supplementary Material 12 [file 12870_2024_5295_MOESM12_ESM.pdf]

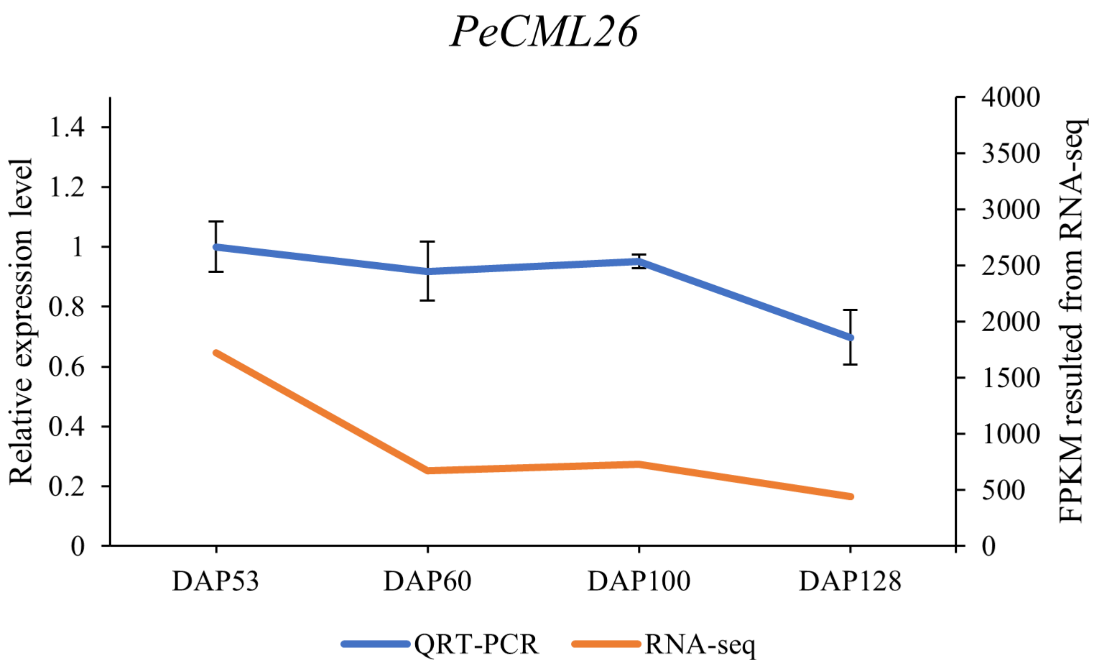

Supplementary Materials Figure S3: The qRT-PCR analysis of *PeCML26* at different stages of passion fruit.
