# Supplementary figures and images for: Genome-wide identification and expression analysis of calmodulin and calmodulin-like genes in passion fruit (Passiflora edulis) and their involvement in flower and fruit development
Source: BMC Plant Biol. 2024 Jul 3;24:626. doi: 10.1186/s12870-024-05295-y (PMC11220982; doi:10.1186/s12870-024-05295-y)

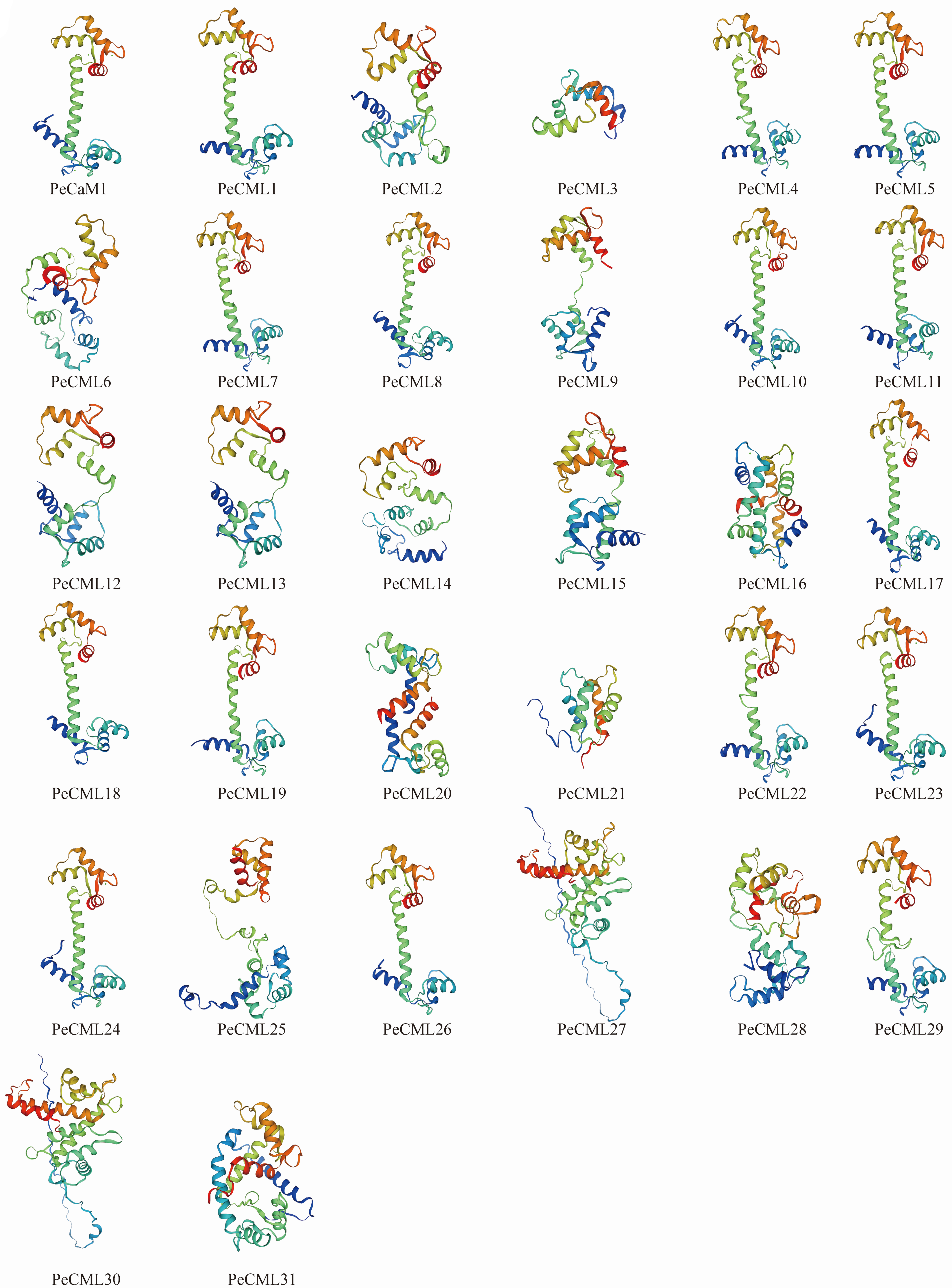

Supplementary Materials Figure S2: Predicted 3D structures of PeCaM/PeCML proteins.

Supplement: Supplementary file 13 — Supplementary Material 13 [file 12870_2024_5295_MOESM13_ESM.pdf]
